# A Quorum-Sensing Inhibitor Strain of *Vibrio alginolyticus* Blocks Qs-Controlled Phenotypes in *Chromobacterium violaceum* and *Pseudomonas aeruginosa*

**DOI:** 10.3390/md17090494

**Published:** 2019-08-24

**Authors:** José Carlos Reina, Ignacio Pérez-Victoria, Jesús Martín, Inmaculada Llamas

**Affiliations:** 1 Department of Microbiology, Faculty of Pharmacy, University of Granada, Campus Universitario Cartuja s/n, 18071 Granada, Spain; 2 MEDINA Foundation, Andalusian Center of Excellence for Research into Innovative Medicines, Health Sciences Technological Park (PTS), Avda. del Conocimiento 34, 18016 Armilla, Granada, Spain; 3 Institute of Biotechnology, Biomedical Research Center (CIBM), University of Granada, 18100 Granada, Spain

**Keywords:** quorum sensing inhibitor, marine bacteria, aquaculture, tyramine, *N*-acetyltyramine, LC–MS, NMR

## Abstract

The cell density-dependent mechanism, quorum sensing (QS), regulates the expression of virulence factors. Its inhibition has been proposed as a promising new strategy to prevent bacterial pathogenicity. In this study, 827 strains from the microbiota of sea anemones and holothurians were screened for their ability to produce quorum-sensing inhibitor (QSI) compounds. The strain M3-10, identified as *Vibrio alginolyticus* by 16S rRNA gene sequencing, as well as ANIb and dDDH analyses, was selected for its high QSI activity. Bioassay-guided fractionation of the cell pellet extract from a fermentation broth of strain M3-10, followed by LC–MS and NMR analyses, revealed tyramine and *N*-acetyltyramine as the active compounds. The QS inhibitory activity of these molecules, which was confirmed using pure commercially available standards, was found to significantly inhibit *Chromobacterium violaceum* ATCC 12472 violacein production and virulence factors, such as pyoverdine production, as well as swarming and twitching motilities, produced by *Pseudomonas aeruginosa* PAO1. This constitutes the first study to screen QSI-producing strains in the microbiota of anemones and holothurians and provides an insight into the use of naturally produced QSI as a possible strategy to combat bacterial infections.

## 1. Introduction

The emergence of antibiotic-resistant pathogenic bacteria has become a major worldwide health concern that threatens the effectiveness of key modern medical treatments of bacterial infections and causes an increasing number of fatalities. This situation highlights the urgency of responding to the demand for new drugs and treatment targets [1]. In recent years, considerable research has consequently focused on developing novel strategies to control bacterial diseases. 

The cell density-dependent mechanism, quorum sensing (QS), controls the expression of virulence genes, including virulence factors and exoenzymes, antibiotic production, as well as exopolysaccharide and biofilm formation, in many bacterial pathogens [2,3,4,5,6,7]. This cell-to-cell communication coordinates the expression of certain genes in response to the accumulation and recognition of threshold concentrations of signal molecules in the surrounding medium, in addition to increases in cell density [8,9]. *N*-Acylhomoserine lactones (AHLs) produced by Gram-negative bacteria, oligopeptides produced by Gram-positive bacteria, and the furanosylborate diester (AI-2) used for interspecies communication are the most studied QS signal molecules [10,11].

Given the regulation by QS of virulence gene expression in many pathogens and its essential role in establishing bacterial diseases, silencing the expression of these genes has been proposed as a new eco-friendly strategy to treat bacterial infections [12,13,14,15,16,17,18,19]. This antivirulence approach involves attenuating bacterial infections rather than killing bacteria or inhibiting bacterial growth. As QS-regulated genes are generally not essential for bacteria, this strategy is not expected to cause selective pressure on the pathogens, causing less bacterial resistance [20].

AHL-based QS systems have been described to be disrupted by various mechanisms. These include the synthesis of compounds known as quorum-sensing inhibitors (QSIs), which act as AHL antagonists and interfere with signal molecule detection [21,22]. Another mechanism, known as quorum quenching (QQ), consists of AHL enzymatic inactivation, mainly through the production of acylases, AHL lactonases, and AHL oxidoreductases [23]. Numerous reports have demonstrated anti-QS activity in a wide range of locations, including plant extracts [24,25,26,27], fungi [28,29], and host-associated bacteria [16,30,31].

Marine-derived microorganisms, which produce numerous active secondary metabolites, are considered an important resource in the search for novel anti-QS compounds [32,33,34,35,36,37]. These include marine invertebrate symbiotic microorganisms, considered to be an underexplored source of new bioactive molecules [38,39,40,41].

In this study, we screened a collection of 827 bacterial strains previously isolated from the microbiota of marine invertebrates such as sea anemones and holothurians [42] because of their ability to produce QSI compounds. One of these isolated bacteria was selected, taxonomically identified, and further assayed in vitro for its anti-QS potential. The active QSI compounds from this bacterium were identified and characterized using LC–MS and NMR analyses following a bioassay-guided fractionation of the bacterial fermentation broth. To our knowledge, this is the first study to screen marine invertebrate microbiota belonging to the phyla *Cnidaria* and *Echinodermata* for the presence of QSI-producing bacteria. We also demonstrate, for the first time, that tyramine and *N*-acetyltyramine act as QSIs.

## 2. Results

### 2.1. Isolation and Taxonomical Identification of QSI-producing Bacteria from Anemones and Holothurians

In a previous study, the 827 strains tested in this paper were isolated from the microbiota and faeces of the invertebrate marine species *Anemonia sulcata*, *Actinia equina*, *Holothuria tubulosa*, and *Holothuria forskali* [42]. QS inhibitory capacity was firstly tested in 96-well microtitre plates using the *Chromobacterium violaceum* ATCC 12472 biosensor, a strain which produces the purple pigment violacein when its QS system is not blocked. Sixty out of the 827 isolates (7.26%) were found to inhibit violacein production, thus indicating anti-QS potential.

The QSI activity of the 60 strains selected was secondly tested in an agar-plate diffusion assay using the same biosensor *C. violaceum* ATCC 12472, which showed that 23 strains partially or totally inhibited the production of violacein (Table 1). These 23 QSI-producing bacteria were then taxonomically identified based on their partial 16S rRNA gene sequences (approximately 800 bp) (Table 1). All sequences were highly homologous to those of certain species of the genus *Vibrio*, particularly *V. neocaledonicus* and *V. alginolyticus,* as compared to reference 16S rRNA sequences obtained from the GenBank database using the BLASTn search option as well as the EzBioCloud website.

The 23 QSI-producing strains selected were tested to discard those that inhibit violacein production by the biosensor strain due to the production of AHL-degrading enzymes. Thus, QQ activity was tested against the synthetic compounds C6-HSL and C10-HSL, and the remaining AHLs were detected by a well diffusion agar-plate assay with *C. violaceum* CV026 and *C. violaceum* VIR07, respectively. None of the 23 strains were found to degrade the AHLs (data not shown). 

The five most active strains (M3-10, M4-31, M4-119, M5-47, and M6-50) were selected (Table 1). To confirm their QSI activity, extracts from the cell pellet and the supernatant from each strain fermentation broth were obtained as described in the Materials and Methods section. The strains were then tested for their ability to inhibit violacein production in *C. violaceum* ATCC 12472 at a final extract concentration of 0.8 mg/mL. On the basis of the results shown in Table 2, the M3-10 strain, which showed the highest QSI activity in both cell pellet and supernatant extracts, was finally selected for further study.

### 2.2. Effect of Cell Pellet Extract from Strain M3-10 on C. Violaceum ATCC 12472 Violacein Production, Pyoverdine Production, Biofilm Formation, and Pseudomonas aeruginosa PAO1 Motility

The cell pellet extract of the selected QSI-producing *V. alginolyticus* M3-10 strain was firstly tested for its inhibitory effect on the growth of *P. aeruginosa* PAO1 and *C. violaceum* ATCC 12472. The growth for each bacterium was unaffected in the presence of the cell pellet extract at a final concentration of 0.8 mg/mL, as the number of colony-forming units (CFUs)/mL after a 24 h incubation period remained the same in the presence or absence of the cell pellet extract (data not shown). The effect of the cell pellet extract at the same concentration on the production of different QS-regulated cellular functions in both bacteria was also tested. The results indicated that the addition of the cell pellet extract of strain M3-10 reduced violacein production by 66% in *C. violaceum* ATCC 12472, as well as virulence factors such as biofilm formation (28%), pyoverdine production (26%), and *P. aeruginosa* PAO1 motility (Table 3). Although differences were observed in the three types of motility, they only were statistically significant for the swimming motility.

### 2.3. Identification of QSI Compounds from V. alginolyticus M3-10

**Figure 1:**
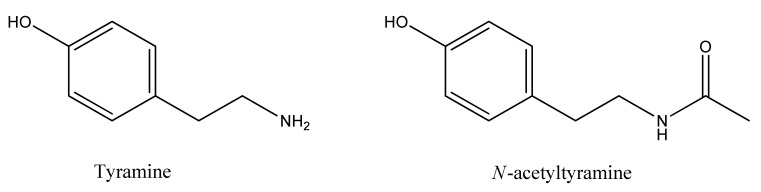
Chemical structure of QSI compounds tyramine and *N*-acetyltyramine produced by *V. alginolyticus* M3-10.

To identify the QSI compounds in metabolites produced by *V. alginolyticus* M3-10, a bioassay-guided fractionation strategy was used. To this end, the cell pellet extract obtained from the 3 L fermentation broth of this bacterium was first fractionated by low-resolution reversed-phase flash chromatography (see Supplementary Figure S1) to generate 25 fractions. QSI activity was localized on two contiguous fractions (12th and 13th), which were then pooled and further fractionated by reversed-phase semipreparative HPLC (see Supplementary Figure S2) to generate 80 fractions. QSI activity was centered on the 13th fraction, and minor activity was likewise detected in its contiguous fractions (12th and 14th). These three HPLC fractions were each analyzed by LC–DAD–HRMS and NMR (^1^H, COSY, TOCSY, HSQC, and HMBC spectra) to identify any known compounds. Dereplication against our in-house databases [43,44] identified the presence of both tyramine and *N*-acetyltyramine (Figure 1) in each of the three bioactive HPLC fractions (see Supplementary Figure S3). The NMR signals reported for these two related compounds [45,46] were identified in the ^1^H NMR spectra of the three fractions (see Supplementary Figures S4–S6). Additionally, direct comparison with the ^1^H and HSQC NMR spectra of tyramine and *N*-acetyltyramine standards, acquired in the same spectrometer, further confirmed the unambiguous identification of the two compounds (Supplementary Figures S7–S13 and Supplementary Tables S1 and S2). Along with the tyramine-related metabolites, two diketopiperazines were also identified, but only in one or two of the three bioactive fractions, after analysis of 2D NMR spectra (data not shown) and comparison with reported data, namely, cyclo-(l-Leu-l-*trans*-4-hydroxyproline or its enantiomer) in the 12th fraction [47] and cyclo-(d-Phe-l-*trans*-4-hydroxyproline or its enantiomer) in the 13th and 14th fractions [48] in similar amounts. The occurrence of these diketopiperazines in just one or two of the three bioactive fractions, along with their relative amounts (see Supplementary Table S3), automatically ruled them out as responsible for the QSI activity displayed by these HPLC fractions. Thus, either tyramine or *N*-acetyltyramine, or both related compounds, were proposed as the QSI compounds produced by the *V. alginolyticus* M3-10 strain. Taking advantage of their commercial availability, a pure standard of each compound was purchased in order to evaluate their QSI activity and to confirm their status as the key QSI compounds produced by *V. alginolyticus* M3-10.

### 2.4. Evaluation of QSI Activity of Tyramine and N-acetyltyramine Standards

Pure commercial tyramine and *N*-acetyltyramine standards were each tested for QSI activity in the production of different QS-controlled cellular functions in *C. violaceum* ATCC 12472 and *P. aeruginosa* PAO1. 

**Figure 2:**
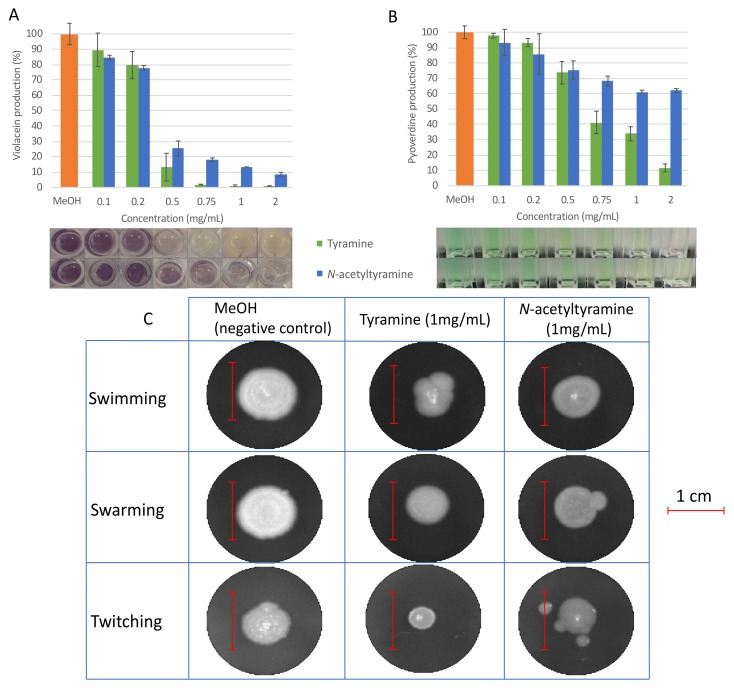
QSI activity of tyramine and *N*-acetyltyramine. Effect of tyramine (green bars) and *N*-acetyltyramine (blue bars) on violacein production in C. *violaceum* ATCC 12472 (**A**); effect of tyramine (green bars) and *N*-acetyltyramine (blue bars) on pyoverdine production (**B**) and on swimming, swarming, and twitching motilities (**C**) in *P. aeruginosa* PAO1. Values are presented as mean ± SD, *n* = 3. The scale bar represents one centimeter. The percentage of reduction was calculated based on the area of expansion that was obtained analyzing the image with ImageJ.

Both molecules were capable of inhibiting more than 85% (99% ± 0.6% in the case of tyramine and 86.7% ± 0.1% for *N*-acetyltyramine) of violacein production in *C. violaceum* ATCC 12472 when used at a final concentration of 1 mg/mL, while over 75% of violacein production (86.9% ± 9% for tyramine and 75% ± 4.9% for *N*-acetyltyramine) was inhibited at 0.5 mg/mL (Figure 2). As shown in Figure 2, tyramine was slightly more active than *N*-aceyltyramine. In all cases, the reduction in violacein corresponded to that in the control strain, which grew in the same amount of methanol (prior to evaporation). Bacterial growth was tested in the absence and presence of different concentrations of tyramine and *N*-acetyltyramine by colony counting, and no differences were observed up to a final concentration of 1 mg/mL, which ruled out a growth inhibition mechanism as responsible for the reduction observed in violacein production (Supplementary Figure S14). 

Regarding the effect of tyramine and *N*-acetyltyramine on *P. aeruginosa* PAO1 QS-related phenotypes, pyoverdine production and motility were tested in the presence of different concentrations of each compound. In all cases, the strain was grown in the same amount of methanol (previously evaporated). The results demonstrated that tyramine was capable of reducing pyoverdine production by 65% (±4.6%), as well as swarming and twitching motilities by 50% (±2.8% and 56% (±6.2%), respectively, at a final concentration of 1 mg/mL. *N*-acetyltyramine inhibited pyoverdine production by 40% (±1.1%) and swarming motility by 40% (±3.6%) at the same final concentration, but no effect was produced on twitching motility. Tyramine was also capable of reducing swimming motility by 41% (±3.6%), whereas *N*-acetyltyramine reduced swimming motility by 23% (±4.1%) (Figure 2C). No inhibitory effect on pathogen growth was observed at these concentrations (Supplementary Figure S14). As in the violacein test, tyramine was more effective than *N*-acetyltyramine in all cases.

### 2.5. Whole-Genome Analysis of V. alginolyticus M3-10

The DNA of the selected QSI-producing strain was sequenced using Illumina HiSeq 2500 (Illumina Inc, San Diego, CA, USA), yielding a total of 7,513,914 reads. The sequences were trimmed with bbDUK v 37.82 (DOE Joint Genome Institute, Walnut Creek, CA, USA) to eliminate contaminants, adapters, and low-quality bases (https://jgi.doe.gov/data-and-tools/bbtools/). The sequences were then assembled to obtain a genome with a total of 70 contigs measuring 5,1 Mb, with an approximate coverage of 160X. This genome was deposited in NCBI (accession number SEYX00000000).

**Figure 3:**
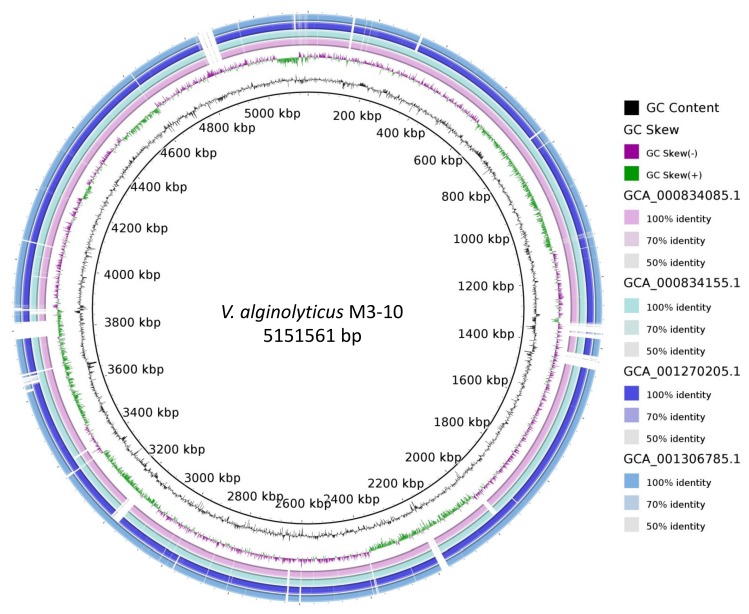
BRIG diagram of chromosomal sequences of the most closely related strains to *V. alginolyticus* M3-10, with this strain as a reference. The gaps in the diagram indicate areas of the genome present in strain M3-10 but absent in the others.

As *V. alginolyticus* was the closest related species to strain M3-10, ANIb was calculated between the M3-10 genome and each of the 38 different *V. alginolyticus* strains in NCBI. Since the ANIb for three of these genomes resulted in values below 95%, the cutoff proposed for the same species [49], they were removed from the analysis. The results of the ANIb analysis are available in Supplementary Table S4, which shows that the maximum similarity of strain M3-10 to any of the other *V. alginolyticus* strains was 98.6%, thus demonstrating how this value varies. A neighbour-joining distance clustering tree based on the ANI values is also available in Supplementary Figure S15, with the M3-10 strain grouped in a different branch of the tree together with two other *V. alginolyticus* strains. Using BRIG software, the M3-10 genome was compared to genomes of the most closely related strains in the clustering tree (see Supplementary Figure S15) to show the differences between strains, with zones in the M3-10 strain not present in related strains (Figure 3).

Tyramine, a metabolic product of tyrosine, is synthetized through tyrosine decarboxylation by tyrosine decarboxylases. A BLASTp search was carried out to find tyrosine decarboxylase homologues. Interestingly, two proteins were found to have high similarity to known tyrosine decarboxylases (with e-values below 1e-30). The encoding genes (locus tags EWT61_07200 and EWT61_20070) were annotated as putative pyridoxal-dependent aspartate 1-decarboxylase and aminotransferase class III-fold pyridoxal phosphate-dependent, respectively. These two proteins have homologies with multiple proteins in the tyrosine decarboxylase custom database. For instance, PRJNA521164:EWT61_07200 shows 26% identity with the tyrosine decarboxylase protein of *Lactobacillus curvatus* (BAE02559.1), whereas PRJNA521164:EWT61_20070 shares 26% of its amino acids with *Pseudomonas entomophila* (BAI67125.1) tyrosine decarboxylase. Moreover, after a Conserved Domains search, both proteins were found to contain the “DOPA_deC-like domain” (e-value 8.31e-70 for PRJNA521164:EWT61_07200 and 2.38e-115 for PRJNA521164:EWT61_20070) commonly found in tyrosine decarboxylases. Furthermore, the two proteins also contained the conserved “beta-eliminating lyase domain”, also typically found in amino acid decarboxylases. 

## 3. Discussion

Messing with quorum sensing has been proposed as a novel promising alternative to the use of antibiotics [12]. Since the discovery of the first QQ enzyme [50], numerous studies have been published in which QQ enzymes are used to control infections produced by human pathogens [51,52] as well as infections in aquaculture [53,54,55,56,57] and agriculture [58,59]. Nevertheless, the vast majority of these studies focus on AHL degradation by QQ enzymes, while only a few deal with non-enzymatic QS inhibition mechanisms or QSI compound production [24,26,60,61].

The marine environment is considered to be an underexplored and untapped source of molecules capable of inhibiting QS systems [17,62,63]. The first QSI compound to be described was a halogenated furanone produced by the red marine alga *Delisea pulchra* [22]. The addition of this compound has been reported to protect both fish and shrimp against vibriosis [64]. Later, numerous extracts from marine invertebrates were shown to exhibit QSI activity [33,61,65,66]. Some bioactive molecules, such as antimicrobial peptides, have been found in invertebrates belonging to the phylum *Echinodermata* [67,68].

Little is known about the production of QSI in the marine symbiotic bacteria of invertebrates. Some bacteria associated with corals have been reported to produce compounds, such as rhodamine isothiocyanate, that inhibit QS in *C. violaceum* [60,69]. Although corals belong to the phylum *Cnidaria*, the present study is, to our knowledge, the first to find QSI compounds in the microbiota associated with sea anemones and holothurians. 

In this study, 23 strains were selected as QSI-producing bacteria based on their capacity to inhibit the QS system of *C. violaceum* ATCC 12472. Interestingly, all these strains belong to the genus *Vibrio*, in which the production of QSI compounds has been described [60,70]. M3-10, identified as a *V. alginolyticus* strain by sequencing its 16s rRNA gene, by whole-genome sequencing, and by ANIb comparison, was again selected, being the most active strain. It belongs to the species *V. alginolyticus* but differs genomically from other strains of the same species. Cell pellet and supernatant extracts were obtained and prepared at the same concentration (0.8 mg/mL). QSI activity was found in both extracts, although it was higher in the cell pellet extract reflecting a higher concentration of QSI compounds in the cells than in the supernatant. This result suggests the requirement for a minimum intracellular threshold concentration of the QSIs to be secreted into the extracellular medium via a passive diffusion mechanism (i. e., mediated by a porin) or an active secretion (i.e., mediated by a transporter) in order to be active. The cell pellet extract from *V. alginolyticus* M3-10, which is capable of inhibiting more than 50% of the violacein production of *C. violaceum* ATCC 12472, was also found to reduce the production of several virulence factors of *P. aeruginosa* PAO1, such as biofilm formation, pyoverdine production, and motility, thus potentially reducing *P. aeruginosa* PAO1 virulence. Interestingly, although the three types of motility (swimming, swarming, and twitching) were reduced in the presence of the cell pellet, only the reduction in swimming motility was statistically significant in our assays after four replicates. *P. aeruginosa* is the most important opportunistic pathogen and the main cause of nosocomial infections [71]. QS is well known to play a crucial role in controlling the virulence of this major pathogen [72], and interference with its QS systems has been proposed as a novel approach in the search for alternatives to the use of effective antibiotics against this pathogen [73].

The whole broth was extracted because our experiments showed that the activity, although higher in the cell pellet, was present in both the cell pellet and the supernatant. The bioassay-guided fractionation of the whole-broth extract of *V. alginolyticus* M3-10 as well as chemical LC–DAD–HRMS and NMR analyses revealed the presence of tyramine and *N*-acetyltyramine as active molecules capable of inhibiting the QS of *C. violaceum* ATCC 12472. The addition of these molecules affected virulence factors produced by *P. aeruginosa* PAO1, lowering pyoverdine production and reducing swimming, swarming, and twitching motilities, with tyramine being more active than *N*-acetyltyramine. Similarly, the molecule hordenine, also a tyramine derivative, has been found to be capable of inhibiting QS in *P. aeruginosa* [74] and *Serratia marcescens* [75] when used in concentrations from 0.5 mg/ml up to 1 mg/mL. This is in line with the findings presented in this study, in which tyramine and *N*-acetyltyramine were found to inhibit violacein production of *C. violaceum* ATCC 12472 at a concentration of 0.5 mg/mL and to inhibit the QS-controlled phenotypes of *P. aeruginosa* PAO1 at a concentration from 1 to 2 mg/mL. Interestingly, a final concentration of 2 mg/mL of both products was toxic to *C. violaceum* but not to *P. aeruginosa*. Other molecules associated with tyramine and *N*-acetyltyramine, such as N-(2’-phenylethyl)-isobutyramide, 3-methyl-N-(2’-phenylethyl)-butyramide [76], 4-(2-hydroxyethyl)-phenol, and tyrosol acetate [77], have also been found to be capable of inhibiting QS-mediated phenotypes, which corroborates our finding that tyramine and *N*-acetyltyramine are QS-inhibiting molecules.

Tyramine, a product of amino acid catabolism found in fish [78] and cheese [79], is produced by different bacteria in a strain-dependent manner [80]. Its synthesis depends on pyridoxal phosphate-dependent decarboxylases [81] found in tyramine-producing Gram-positive [81] and Gram-negative bacteria such as those belonging to the *Enterobacteriaceae* [82,83] and *Aeromonadaceae* [80] families. In the draft genome of strain M3-10, different amino acid decarboxylases have been found, as well as pyridoxalphosphate-dependent decarboxylases with high similarity to known tyrosine decarboxylases. Moreover, homologues motifs are similar to the known motifs of tyrosine decarboxylases [81], thus reinforcing the proposed production of tyramine by M3-10. *N*-acetyltyramine has been identified in an endophytic *Streptomyces* strain [84] and, more recently, in actinomycete *Actinokineospora* sp. [85]. This molecule has actually been reported to contain biological activities that differ from QSI such as radical scavenging [85], anti-platelet aggregation [86], and fungicidal activity [87]. This study is, to our knowledge, the first to demonstrate the presence of QSI in tyramine and *N*-acetyltyramine.

Although some may be considered pathogens to aquatic animals and humans [88], *V. alginolyticus* strains are recognized to be typical coral symbiotic bacteria [60], which are also frequently present in healthy fish, shrimps, and mollusks [89] and whose biotechnological potential has been used for collagenase production in the clinic [90]. With regard to the QSI potential of *V. alginolyticus*, although Wang et al. [91] have reported that the diketopiperazine cyclo (Leu-Pro) produced by a strain of this species isolated from sea anemones is capable of inhibiting mussel fouling, its QSI capacity was not tested. Similarly, phenol 2,4-bis (1,1-dimethylethyl) produced by a *V. alginolyticus* strain has been found to inhibit violacein production in *C. violaceum* as well as QS-regulated virulence factors in *S. marcescens* [92]. Recently, Song et al. (2018) [60] also demonstrated the presence of anti-QS activity in another *V. alginolyticus* strain, isolated from corals, caused by rhodamine isothiocyanate production. Both these findings and our study reinforce the possibility of using *V. alginolyticus* as a potential biotechonological tool in the development of biological alternatives to antibiotics and synthetic antifouling substances through the production of QSI compounds such as tyramine and *N*-acetyltyramine. 

## 4. Materials and Methods 

### 4.1. Bacterial Strains, Media, Compounds, and Culture Conditions

The 827 strains tested in this study were previously isolated from the microbiota and faeces of marine invertebrates including anemones (*A. sulcata* and *A. equina*) and holothurians (*H. tubulosa* and *H. forskali*). The samples were collected and processed in a previous study at iMARE Natural S.L (http://www.imarenatural.com) aquaculture facilities located in Motril, Granada (36°44′33.4″N 3°31′12.1″W), in southern Spain [42]. All marine strains used were routinely grown at 28 °C in marine broth (MB, BD Difco^®^, Franklin Lakes, NJ, USA). The human-related pathogen *P. aeruginosa* PAO1 was cultured in LB medium at 37 °C. *C. violaceum* ATCC 12472 [93], *C. violaceum* CV026 [94], and *C. violaceum* VIR07 [93] were grown at 28 °C in LB medium. In the case of strains CV026 and VIR07, the medium was supplemented with kanamycin (50 µg mL^−1^). The synthetic AHLs used were C6-HSL (*N*-hexanoyl-dl-homoserine lactone) and C10-HSL (*N*-decanoyl-dl-homoserine lactone) (Sigma^®^, Madrid, Spain).

### 4.2. Screening for QS Inhibition Activity

The potential QS inhibition activity of the 827 isolates was first analyzed in 96-well microtitre plates by testing interference with the QS system of *C. violaceum* ATCC 12472. Each bacterial strain was grown in 200 µL of MB medium. After overnight incubation at 28 °C, 20 µL of cultures was transferred to a new microtitre plate with a layer containing 200 µl LB agar (0.7% w/v) previously inoculated with the biosensor *C. violaceum* ATCC 12472. The microplates were incubated for 24 h at 28 °C to check for the absence of violacein pigment production. 

A second assay was performed with the selected strains using a diffusion agar-plate technique. For the test, 20 µL of 24 h culture of each strain was deposited on the surface of a Petri dish, in which the biosensor strain *C. violaceum* ATCC 12472 was previously extended. After 24 h of incubation, the strains showing a partially transparent halo of violacein inhibition, distinct from that indicating antibiosis activity, were selected and assayed three times.

AHL-degrading activity against C6-HSL and C10-HSL was tested in a well diffusion agar-plate assay, as described elsewhere [57,95], which was performed twice. Briefly, 24 h cultures of the selected strains were supplemented with 10 μM C6-HSL or C10-HSL. After 24 h of incubation, the remaining AHLs were detected using the biosensor strains *C. violaceum* CV026 and C. *violaceum* VIR07, respectively. 

### 4.3. Preparation of Methanolic Extracts of QSI-producing Bacteria

Methanolic extracts were prepared according to a method adapted from Saurav et al. and Teasdale et al. [34,66]. 

The selected QSI-producing strains were grown at 28 °C for 5 days in 20 mL of MB medium. The fermentation broth of each bacterium was centrifuged, and the supernatant and cell pellet were separated. The cell pellet was then resuspended in 25 mL of MeOH, sonicated for 15 min in a water bath at room temperature, and agitated for an additional 30 min. After centrifugation, the supernatant was evaporated to generate the corresponding extract and then resuspended in methanol at a concentration of 0.8 mg/mL. In a similar manner, the aqueous supernatant of each bacterial culture was extracted three times with ethyl acetate, and the organic phase was evaporated to generate the corresponding extract, which was finally resuspended in methanol at 0.8 mg/mL. 

### 4.4. Violacein Production Assay

The inhibition of the pigment violacein production by *C. violaceum* ATCC 12472 was analyzed following a protocol described elsewhere [26]. Briefly, 1 mL of a 24 h culture of *C. violaceum* ATCC 12472, grown in the presence of 0.8 mg/mL of each extract (cell pellet or supernatant), was centrifuged, and the supernatant was discarded. Violacein in the cell pellet was extracted with DMSO and centrifuged; the absorbance of the extract was measured at 585 nm. This assay was repeated six times. As a control, *C. violaceum* ATCC 12472 was grown in the presence of the same amount of evaporated methanol without the extract. To check the effect of the presence of each QSI in the growth of the biosensor, a colony count was carried out in each assay. 

### 4.5. Pseudomonas Virulence Factor Tests: Pyoverdine, Biofilm, and Motility

To assess the ability of the selected QSI extracts to affect the production and development of known virulence factors in *P. aeruginosa* PAO1, pyoverdine production, biofilm formation, and motility assays were performed. The pyoverdine assay was carried out as described by Ren et al. (2005) [96]. Briefly, a 1:1000 dilution of a 24 h culture of PAO1 was cultured in minimal iron-deficient medium (6 g/L K_2_HPO_4_, 4 g/L KH_2_PO_4_, 1g/L (NH_4_)_2_SO_4_, 0.2 g/L MgSO_4_ × 7H_2_O, 9.15 g/L sodium succinate) for 24 h in the presence of the cell pellet extract (0.8 mg/mL). After 24 h of incubation, bacterial growth was measured by absorbance at 600 nm. Then, 1 mL of the culture was centrifuged, and the absorbance of the supernatant was measured at 405 nm. Pyoverdine production was calculated as OD_405_/OD_600_. As controls, *P. aeruginosa* PAO1 was grown in the presence of the same amount of methanol without the extract (after its evaporation), as well as without methanol. A colony count was performed to check the effect of the extract on the growth of *P. aeruginosa* PAO1.

The swimming, swarming, and twitching motility assays were performed in six-well plates using 5 mL of each motility medium at 0.3, 0.5, and 1% (*w/v*) agar, respectively [97,98,99]. Briefly, 2 µL of a 24 h *P. aeruginosa* PAO1 culture were inoculated in the center of the well, and growth was examined after 24 h incubation. The media were supplemented with 0.8 mg/mL of the crude methanolic QSI extract and the same volume of MeOH without the extract that was used as a negative control. This assay was repeated four times. The area of expansion was calculated using ImageJ v 1.51 (NIH, USA) [100].

The biofilm formation assay was carried out in 96-well plates according to a modified method described by O′Toole (2011) [101]. Briefly, an overnight culture of PAO1 was inoculated at a ratio of 1:100 in fresh LB medium with and without the selected crude methanolic QSI extract. After 24 h of incubation, cell growth was determined by absorbance at 600 nm. Biofilm was measured by discarding the medium, rinsing the wells with water, and staining the bound cells with 250 μL crystal violet (1%, *w/v*). After 15 min of incubation, the wells were washed with water, and the dyed cells were resuspended in ethanol 95% (*v/v*). Finally, the absorbance was measured at 540 nm. In each experiment, biofilm formation was calculated by subtracting the crystal violet bound to non-inoculated controls. Each assay was performed three times. In all cases, the results were statistically analyzed using Welch′s two-sample t test using RStudio v 1.1.456 (RStudio, Inc, Boston, MA, USA)

### 4.6. Bioassay-Guided Fractionation and Identification of QSI Compounds by LC–DAD–HRMS and NMR

A 5-day 3L fermentation broth of strain M3-10 grown in MB at 28 °C was prepared in order to identify the QSI compounds following bioassay-guided fractionation. The fermentation broth was centrifuged, and the cell pellet obtained was extracted with 600 mL methanol (as described in Section 4.3). Once dried, this methanolic extract was resuspended in 45 mL of methanol and added to the aqueous supernatant from the fermentation broth. The resulting solution was loaded onto a column filled with SP207ss resin (65 g, 32 × 100 mm) and then washed with an equal volume of water. The elution was carried out in a CombiFlash^®^ (Teledyne ISCO, Lincoln, NE, USA) system using a step gradient with increasing acetone content in water (8 mL/min, 20 mL/fraction, 5 min per gradient step, 20% (*v/v*) acetone increment between steps) to generate 25 fractions. These were evaporated to dryness and tested for QSI activity at 0.8 mg/mL. The active fractions were then pooled together and further fractionated by semipreparative HPLC (Atlantis Prep T3, 5 m, 10 × 150 mm, (Waters Corporation, Mildford, MA, USA) 15% (*v/v*) to 25% (*v/v*) acetonitrile in water for 35 min, UV detection at 210 and 280 nm, 3.6 mL/min, 1.8 mL/fraction) on a GILSON GX-281 322H2 LC (Gilson Inc, Middleton, WI, USA) to generate 80 fractions. After drying, the fractions were resuspended in methanol and tested for QSI at three different dilutions to better discriminate the most active fraction. The active fractions were then analyzed by LC–DAD–HRMS, as previously described [44], on a Bruker maXis QTOF mass spectrometer (Bruker Biospin, Fällanden, Switzerland) coupled to an Agilent 1200 LC (Agilent Technologies, Santa Clara, CA, USA). Dereplication against our in-house databases [43,44] was employed to identify the chemical structure of the compounds in the bioactive HPLC fractions. NMR analysis was also carried out after reconstitution of each bioactive fraction in deuterated methanol. NMR spectra were recorded on a Bruker Avance III spectrometer (Bruker Biospin) (500 and 125 MHz for ^1^H and ^13^C NMR, respectively) equipped with a 1.7 mm MicroCryoprobe (Bruker Biospin), using the signal of the residual solvent as internal reference (δ_H_ 3.31 and δ_C_ 49.15 ppm for CD_3_OD). A description of the identification of the QSI compounds, along with the corresponding DAD, HRMS, and NMR spectra, is included in the supporting information (Supplementary Materials Table S1).

### 4.7. QSI inhibition Activity Test of Tyramine and N-acetyltyramine

Tyramine and *N*-acetyltyramine were obtained from Tokyo Chemical Industry Co. Ltd (Tokyo, Japan) and Fluorochem Ltd (Hadfield, UK), respectively. Analysis of violacein and pyoverdine production and motility assays were carried out in the presence and absence of these compounds according to the methodology described above. Tyramine and *N*-acetyltyramine were tested at the final concentrations of 0.1, 0.2, 0.5, 0.75, 1, and 2 mg/mL, except for the motility assays, for which they were tested at 1 mg/mL. All the experiments were carried out in triplicates. To determine whether these compounds affected the growth of *C. violaceum* ATCC 12472 and *P. aeruginosa* PAO1, a colony count was performed in the presence and absence of each compound concentration.

### 4.8. Taxonomic Identification and Genome Analysis of Strain M3-10 

Total genomic DNA from the selected QSI-producing strain was extracted following the technique described elsewhere [102]. The 16s rRNA gene was amplified by PCR using universal primers [103], and the product was purified and sequenced. The bacteria were then identified using the NCBI rRNA 16s database, the BLASTn v 2.7.1 (NIH, USA) search tool [104], and the EzBioCloud server [105] 

The whole genome was sequenced at the STAB Vida facility in Caparica in Portugal (https://www.stabvida.com), with total genomic DNA extracted according to Marmur’s method [106]. After de novo assembly into contigs using SPAdes v 3.11.1 (Center for Algorighmic Biotechnology, St. Petersburg, Russia) [107], the genome was deposited in NCBI and annotated through PGAP v 4.7 (NIH, USA) [108]. The genomic DNA was compared using the ANIb genomic distance ANI-Matrix calculator (Kostas lab, Atlanta, GA, USA) [109], with the 35 annotated *V. alginolyticus* genomes as references. BRIG v 0.95 software (http://brig.sourceforge.net) [110] was used to compare the genomes of the most closely related strains to M3-10 and to generate a ring visualization.

As tyramine is a product of tyrosine [81], in order to evaluate the presence of protein homologues of tyrosine decarboxylase, a local BLASTp v 2.7.1 (NIH, USA) [111] search was performed using a total of 94 tyrosine decarboxylases proteins as queries (see Supplementary Table S5) and all M3-10 proteins as database. The potential protein homologues were then analyzed using an NCBI Conserved Domains Search v 3.17 (NIH, USA) [112,113,114,115], to check for common domains conserved in amino acid decarboxylases. 

## Figures and Tables

**Table 1:** Taxonomic identification of selected quorum-sensing inhibitor (QSI)-producing strains and their ability to inhibit violacein production in *Chromobacterium violaceum* ATCC 12472.

Strain	Identification	Violacein Inhibition Halo (mm)
M1-9	*Vibrio neocaledonicus*	16
M2-99	*Vibrio antiquarius*	13
M3-10	*Vibrio alginolyticus*	18
M3-21	*V. neocaledonicus*	15
M3-78	*V. alginolyticus*	15
M3-99	*V. alginolyticus*	15
M4-31	*V. alginolyticus*	17
M4-35	*V. alginolyticus*	12
M4-116	*V. alginolyticus*	13
M4-117	*V. neocaledonicus*	12
M4-119	*V. neocaledonicus*	16
M4-126	*V. neocaledonicus*	11
M5-23	*V. neocaledonicus*	13
M5-35	*V. neocaledonicus*	16
M5-47	*V. neocaledonicus*	18
M5-50	*V. neocaledonicus*	13
M6-17	*V. neocaledonicus*	13
M6-26	*V. neocaledonicus*	13
M6-31	*V. alginolyticus*	11
M6-39	*V. zhuhaiensis*	14
M6-50	*V. neocaledonicus*	17
M6-66	*V. alginolyticus*	15
M10-18	*V. neocaledonicus*	14

**Table 2:** Violacein inhibition capacity of cell pellet and supernatant extracts of the five most effective strains.

	Extract	Violacein Production (Absorbance at 585 nm)
M3-10	cell pellet	0.034
	supernatant	0.206
M4-31	cell pellet	0.044
	supernatant	0.282
M4-119	cell pellet	0.06
	supernatant	0.276
M5-47	cell pellet	0.044
	supernatant	0.216
M6-50	cell pellet	0.042
	supernatant	0.280
	MeOH (negative control)	0.489

**Table 3:** Determination of quorum-sensing (QS) phenotypes in *Chromobacterium violaceum* ATCC 12472 and *Pseudomonas aeruginosa* PAO1 in the absence and presence of QSI cell pellet extract.

	Mean in the Absence Of Cell Pellet Extract	Mean in the Presence Of Cell Pellet Extract
***C. violaceum* ATCC 12472**		
Violacein (% production)	100 [±10.9]	33.89 * [±17.1]
***P. aeruginosa* PAO1**		
Pyoverdine (% production)	100 [±16.1]	74.02 * [±8]
Biofilm (% production)	100 [±12.7]	71.96 * [±5.9]
Swimming (% motility)	55.6 [±8.1]	42.8 * [±2.5]
Swarming (% motility)	39.46 [±15.3]	27.36 [±9.6]
Twitching (% motility)	39.15 [±13.6]	31.54 [±10.7]

* = *p* < 0.05.

## Supplementary Materials

The following are available online at https://www.mdpi.com/1660-3397/17/9/494/s1, Figure S1: Low resolution flash chromatography after solid phase extraction of the fermentation broth using SP207ss resin, Figure S2: Semipreparative HPLC chromatographic fractionation of the pool of the two active fractions (12 and 13) obtained after the previous low-resolution flash chromatography step, Figure S3: UV-vis (DAD) and HRMS spectra of *N*-acetyltyramine (two upper spectra) and tyramine (two lower spectra) detected after LC-DAD-HRMS analysis of the three semipreparative HPLC fractions which showed QSI activity, Figure S4: 1H NMR spectra of QSI-active HPLC fractions 12 (green), 13 (red) and 14 (blue), (500 MHz, CD3OD), Figure S5: Expansion of Fig. S4 spectra, indicating the common aromatic signals observed in all three spectra, Figure S6: A further expansion of Figure S4 spectra, indicating the common aliphatic signals observed in all three spectra, Figure S7: 1H NMR spectra of QSI-active HPLC fraction 13 (red) and *N*-acetyltyramine standard (blue) (500 MHz, CD3OD), Figure S8: Expansion of spectra in Supplementary Figure S7, indicating the identification of the aromatic signals from *N*-acetyltyramine, Figure S9: A further expansion of the spectra in Supplementary Figure S7, indicating the identification of the aliphatic signals from *N*-acetyltyramine. Figure S10: 1H NMR spectra of QSI-active HPLC fraction 12 (green), the same fraction spiked with pure tyramine (red) and tyramine standard (blue) (500 MHz, CD3OD), Figure S11: HSQC spectra of QSI-active HPLC fraction 12 (upper), *N*-acetyltyramine standard (middle) and tyramine standard (lower), Figure S12: HSQC spectra of QSI-active HPLC fraction 13 (upper), *N*-acetyltyramine standard (middle) and tyramine standard (lower), Figure S13: HSQC spectra of QSI-active HPLC fraction 13 (upper), *N*-acetyltyramine standard (middle) and tyramine standard (lower), Figure S14: Growth of *C. violaceum* ATCC 14472 (A) and *P. aeruginosa* PAO1 (B), Figure S15: Neighbor-Joining distance clustering tree based on the ANI values between M3-10 and the rest of the *Vibrio alginolyticus* genomes available in NCBI, Table S1: NMR assignment (protonated carbons) of tyramine and *N*-acetyltyramine signals observed in the three QSI-active HPLC fractions (500 MHz, CD3OD), Table S2: NMR assignment (protonated carbons) of tyramine and *N*-acetyltyramine standards (500 MHz, CD3OD), Table S3: Relative abundance (estimated by NMR) of tyramine, N-acetyltyramine and diketopiperazines in the three QSI-active HPLC fractions 12, 13 and 14, Table S4: ANIb values of strain M3-10 compared to the other *V. alginolyticus* strains whose genome is available, Table S5: Protein sequence accession numbers for tyrosine decarboxylases used in the BLASTp homologue search, Methods S1: Identification of QSI compounds in active HPLC fractions using LC-DAD-HRMS and NMR analyses.
