# Supplementary material for: A Quorum-Sensing Inhibitor Strain of Vibrio alginolyticus Blocks Qs-Controlled Phenotypes in Chromobacterium violaceum and Pseudomonas aeruginosa
Source: Mar Drugs. 2019 Aug 24;17(9):494. doi: 10.3390/md17090494 (PMC6780304; doi:10.3390/md17090494)
Supplement: Supplementary file 1 [file marinedrugs-17-00494-s001.pdf]

Supplementary Materials:

A quorum-sensing inhibitor strain of *Vibrio alginolyticus* blocks QS-controlled phenotypes in *Chromobacterium violaceum* and *Pseudomonas aeruginosa*

José Carlos Reina[1], Ignacio Pérez-Victoria[2,*], Jesús Martín[2] and Inmaculada Llamas [1,3*]

[1] Department of Microbiology, Faculty of Pharmacy, University of Granada, Campus Universitario Cartuja s/n, 18071 Granada, Spain
[2] MEDINA Foundation, Andalusian Center of Excellence for Research into Innovative Medicines, Health Science Technology Park, Avda. del Conocimiento 34, 18016 Armilla, Granada, Spain.
[3] Institute of Biotechnology, Biomedical Research Center (CIBM), University of Granada, 18100 Granada, Spain

* Correspondence: illamas@ugr.es (I.L.), ignacio.perez-victoria@medinaandalucia.es (I.P.V.)

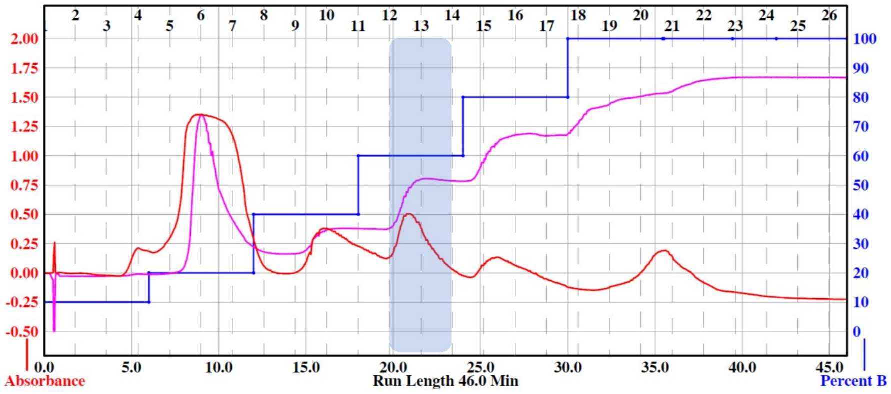

Supplementary Figure S1. Low resolution flash chromatography after solid phase extraction of the fermentation broth using SP207ss resin (Solvent A: water, Solvent B: acetone; Flow rate: 10 mL/min; Red trace: 210 nm, Purple trace: 280 nm). Fractions 12 and 13 displaying QSI activity are highlighted.

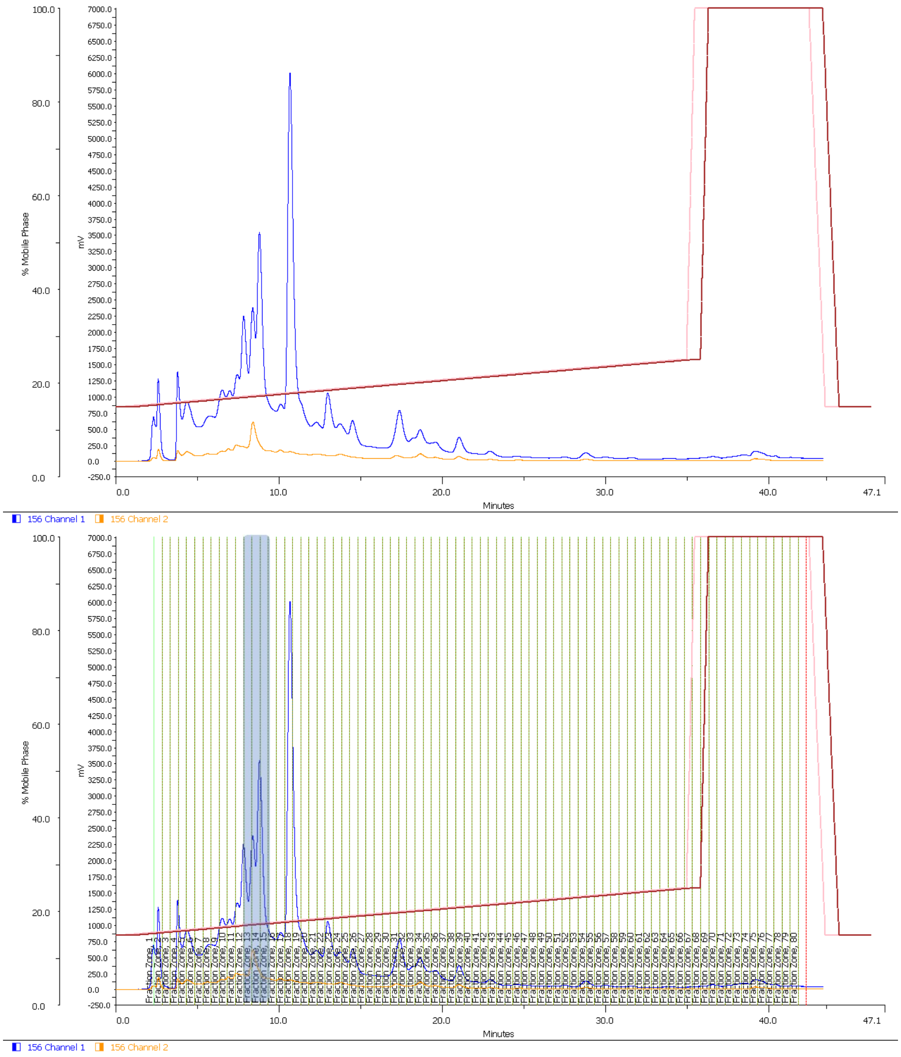

Supplementary Figure S2. Semipreparative HPLC chromatographic fractionation of the pool of the two active fractions (12 and 13) obtained after the previous low-resolution flash chromatography step (Solvent A: water, Solvent B: acetonitrile; Flow rate: 10 mL/min). Fractions 12, 13 and 14 displaying QSI activity are highlighted.

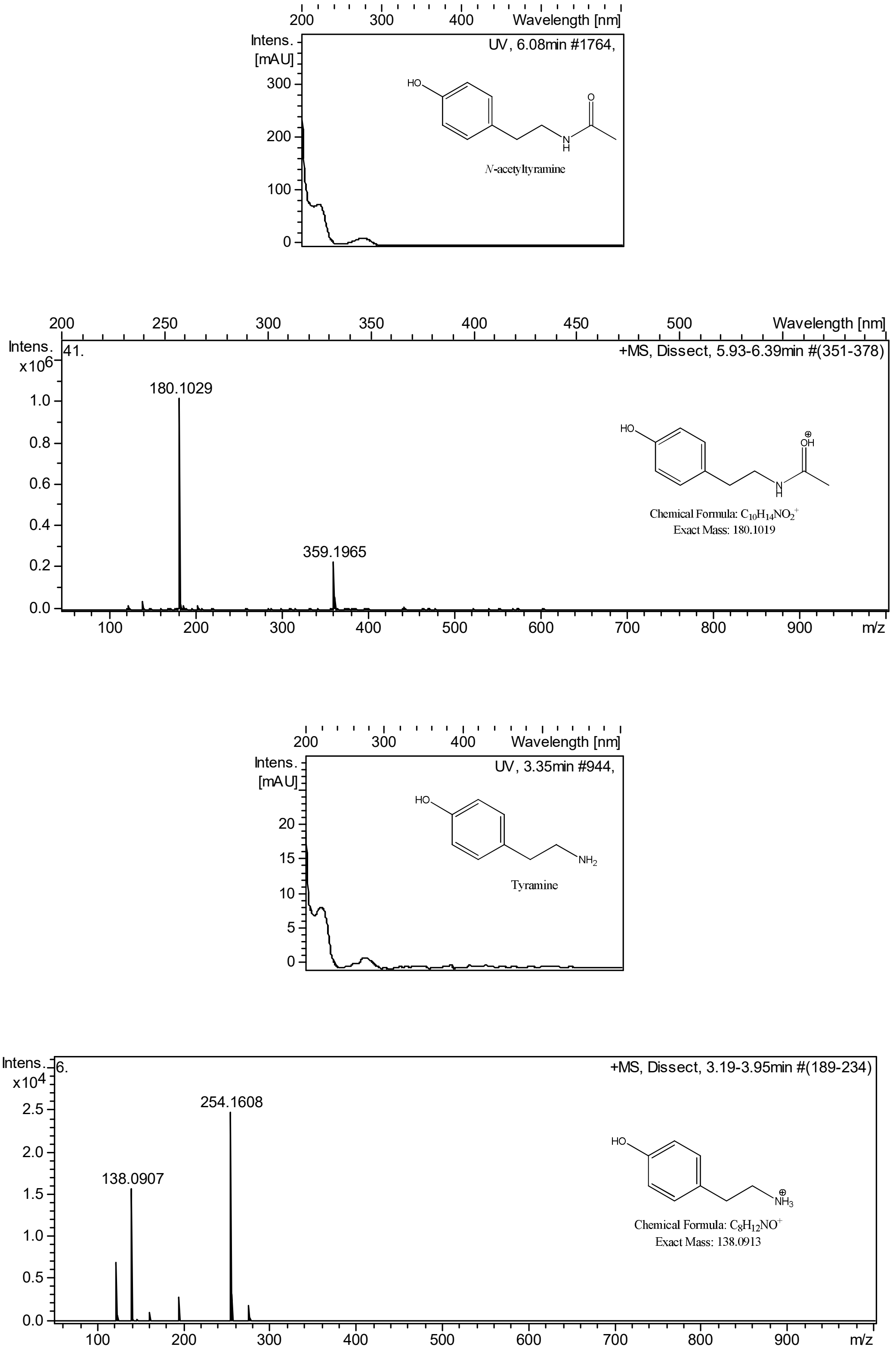

Supplementary Figure S3. UV-vis (DAD) and HRMS spectra of N-acetyltyramine (two upper spectra) and tyramine (two lower spectra) detected after LC-DAD-HRMS analysis of the three semipreparative HPLC fractions which showed QSI activity. The two compounds were observed in the three fractions, whose DAD and HRMS spectra, along with their retention time, were employed for their dereplication using our in-house databases [1,2].

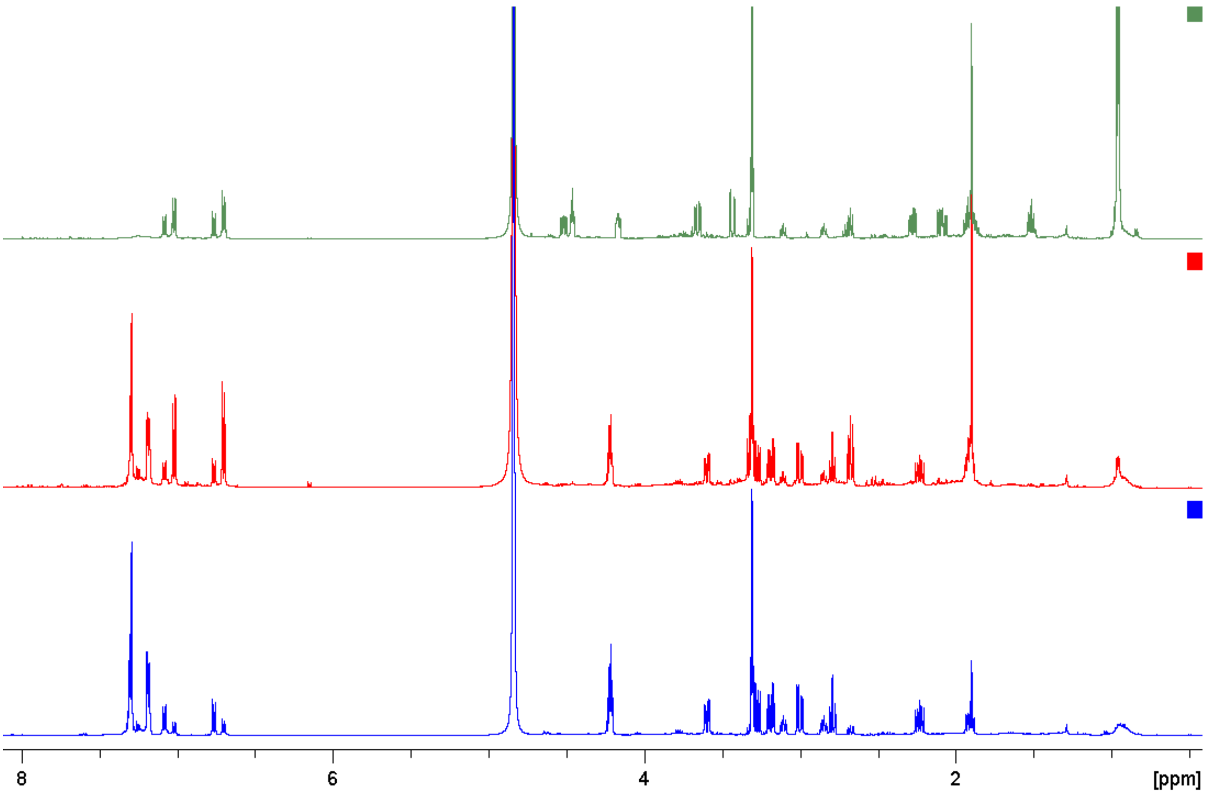

Supplementary Figure S4. 1H NMR spectra of QSI-active HPLC fractions 12 (green), 13 (red) and 14 (blue), (500 MHz, CD3OD).

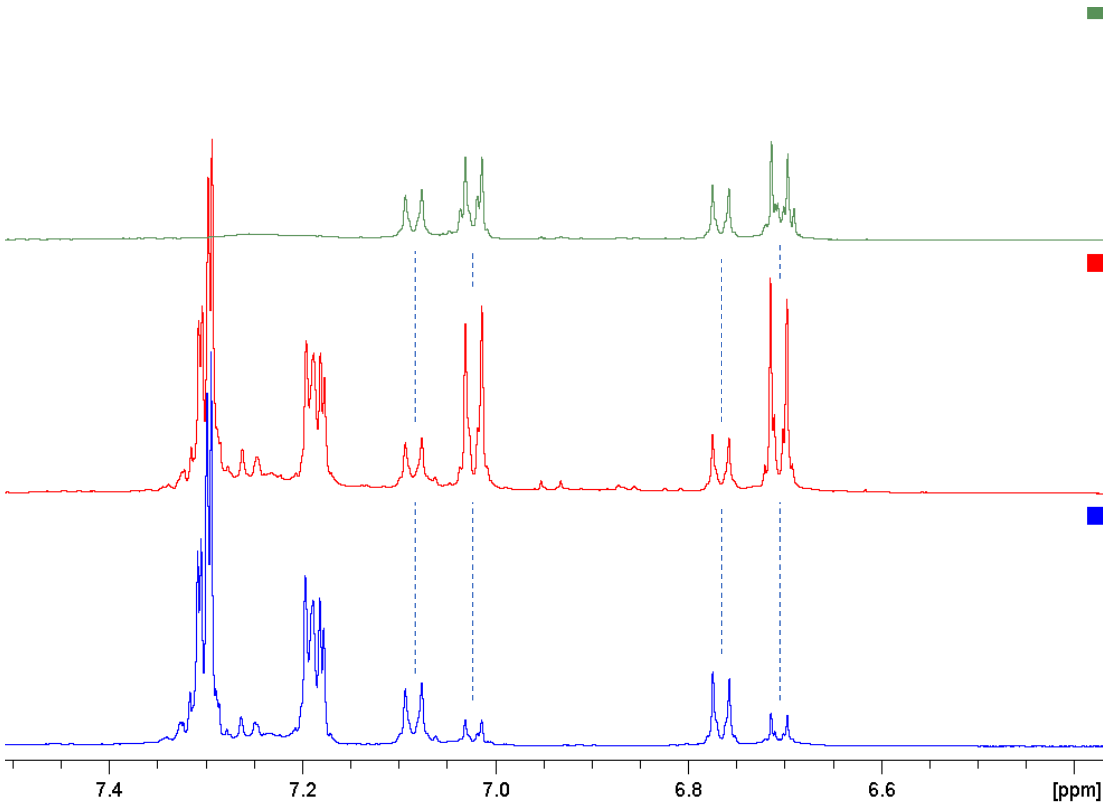

Supplementary Figure S5. Expansion of Fig. S4 spectra, indicating the common aromatic signals observed in all three spectra.

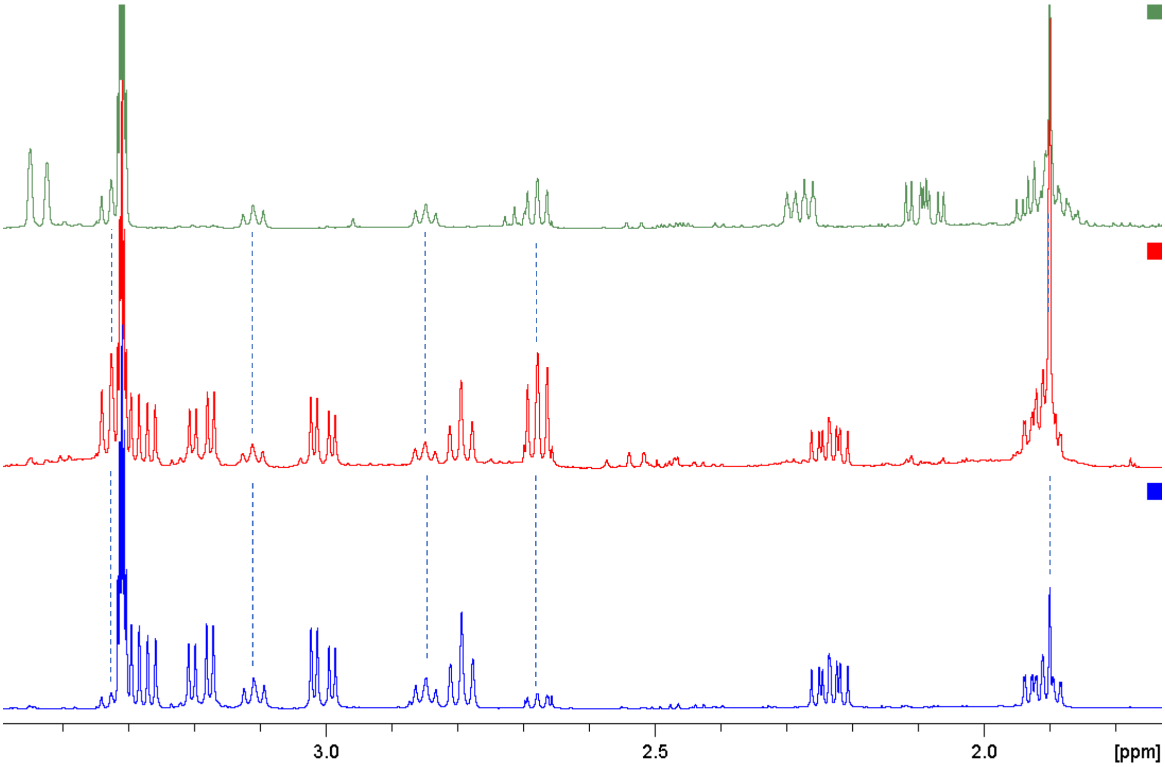

Supplementary Figure S6. A further expansion of Fig. S4 spectra, indicating the common aliphatic signals observed in all three spectra.

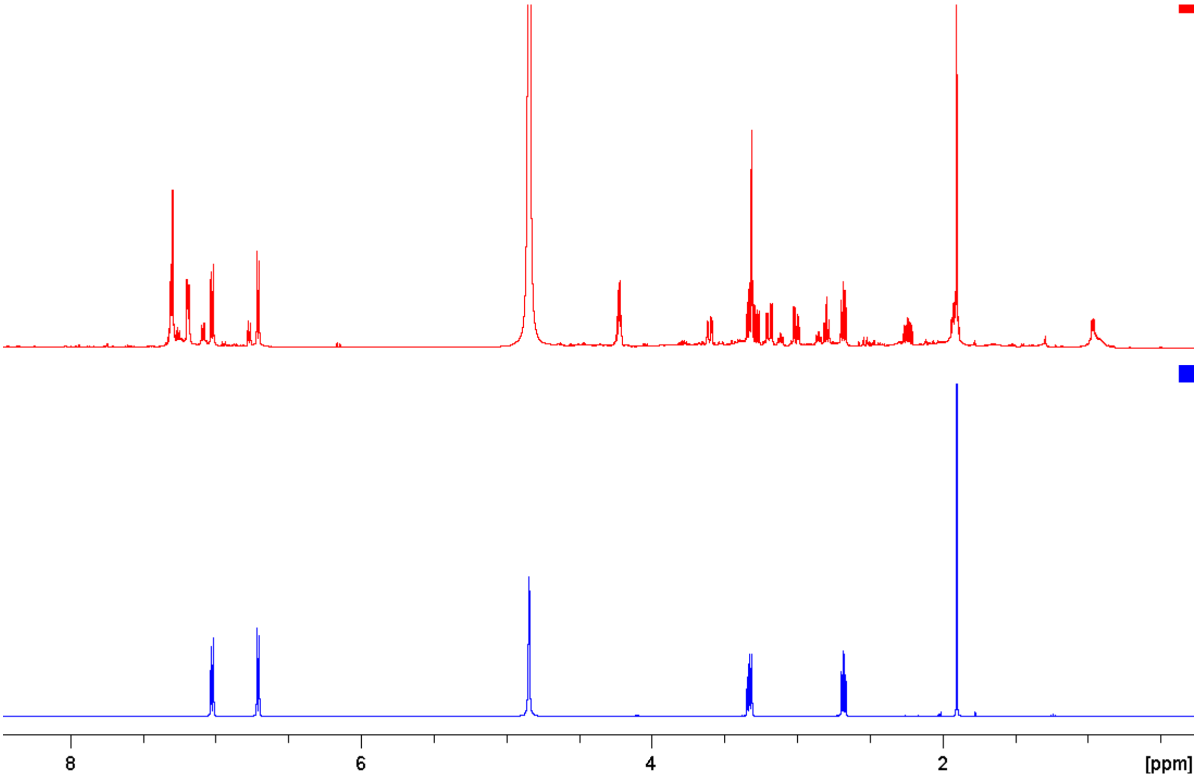

Supplementary Figure S7. 1H NMR spectra of QSI-active HPLC fraction 13 (red) and N-acetyltyramine standard (blue) (500 MHz, CD3OD).

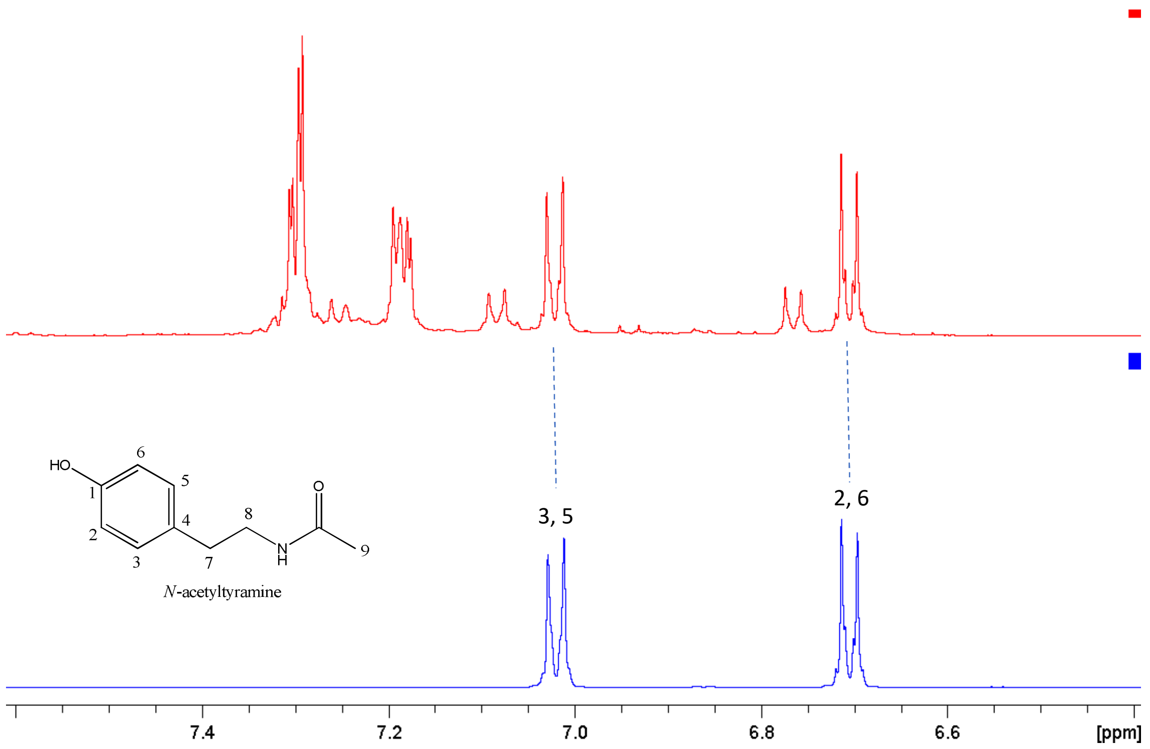

Supplementary Figure S8. Expansion of spectra in Supplementary Figure S7, indicating the identification of the aromatic signals from N-acetyltyramine.

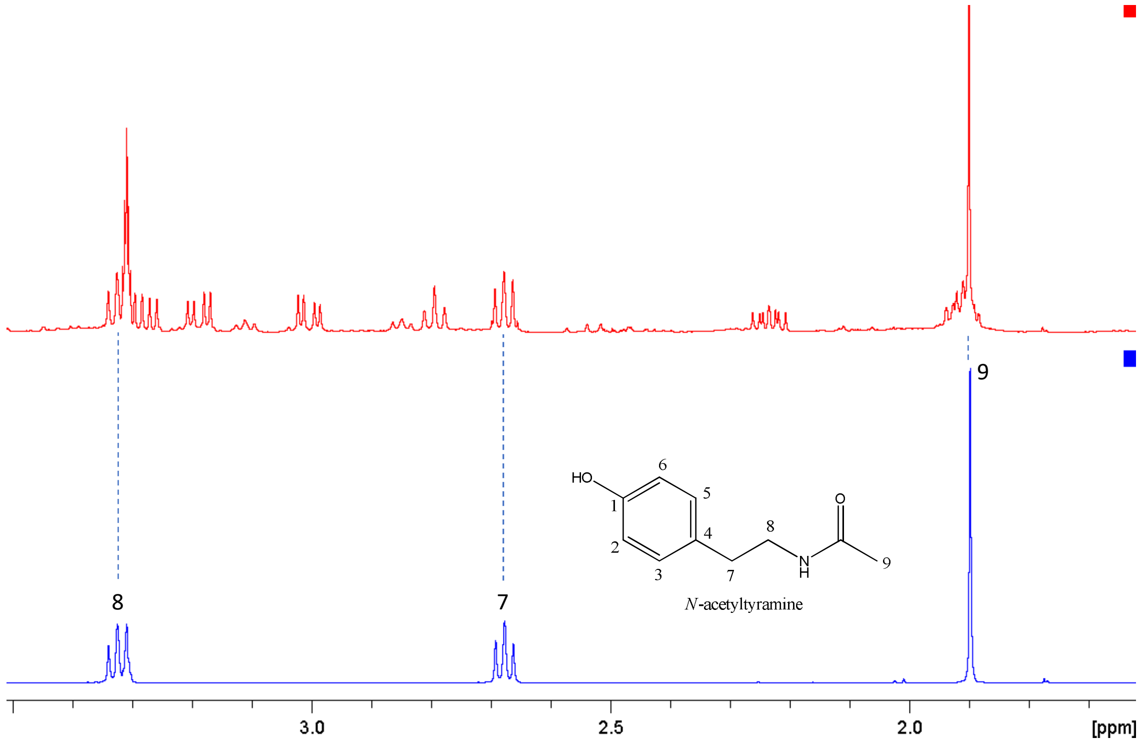

Supplementary Figure S9. A further expansion of the spectra in Supplementary Figure S7, indicating the identification of the aliphatic signals from N-acetyltyramine.

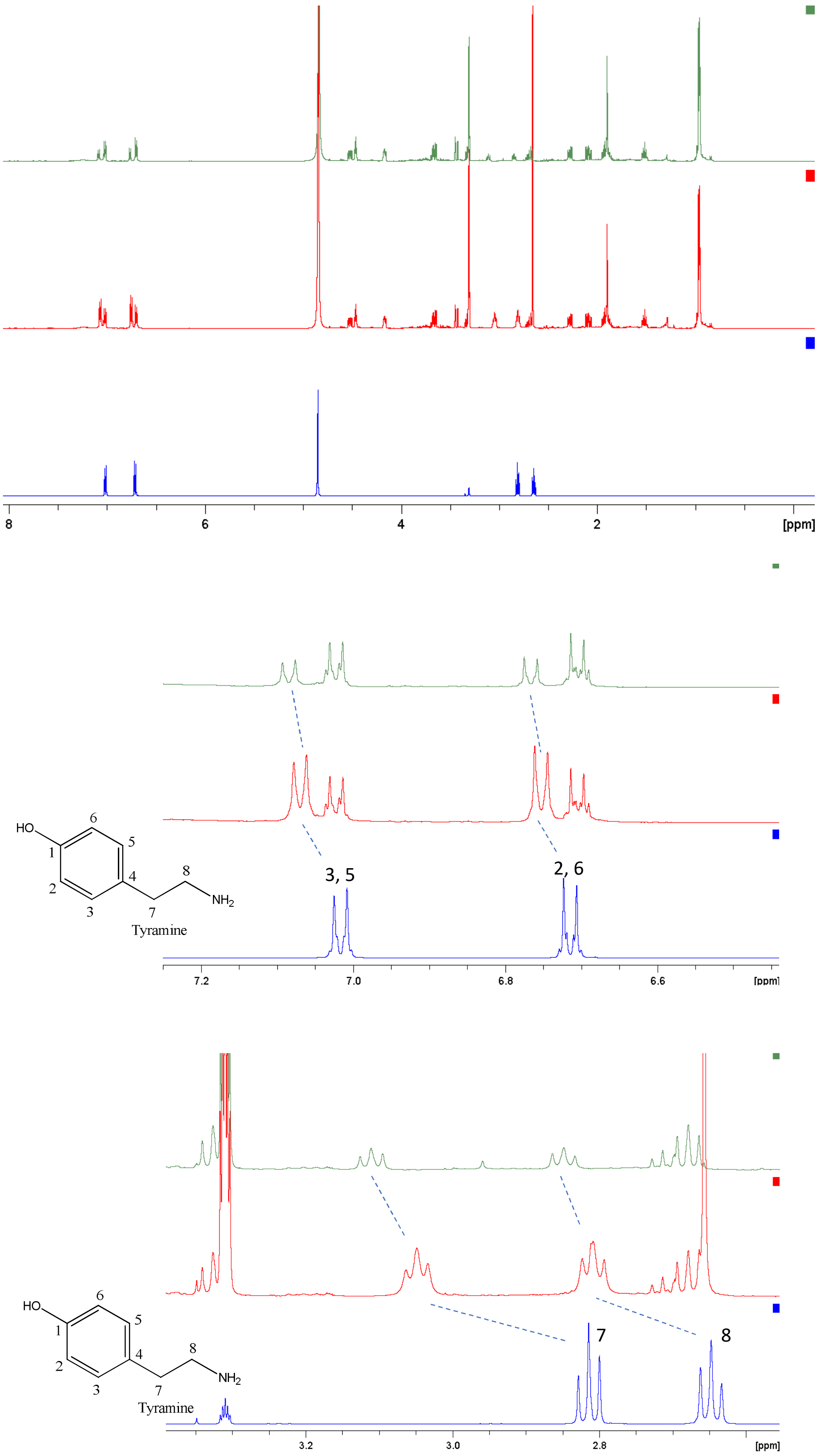

Supplementary Figure S10. 1H NMR spectra of QSI-active HPLC fraction 12 (green), the same fraction spiked with pure tyramine (red) and tyramine standard (blue) (500 MHz, CD3OD). The expansions indicate the identification of the aromatic (first expansion) and aliphatic (second expansion) signals from tyramine.

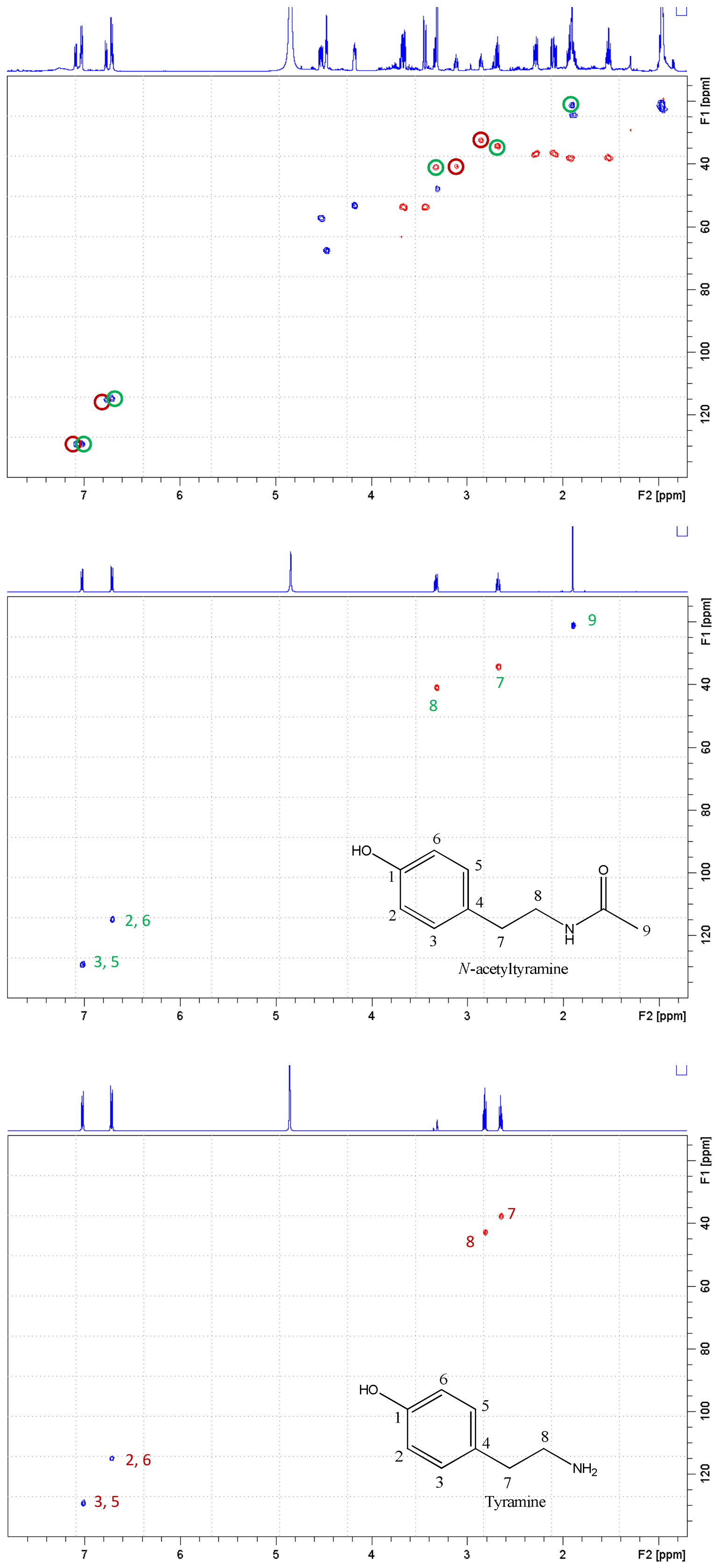

Supplementary Figure S11. HSQC spectra of QSI-active HPLC fraction 12 (upper), N-acetyltyramine standard (middle) and tyramine standard (lower). Signals from these two molecules are highlighted in the upper spectrum, where the non-highlighted signals correspond to cyclo-(L-Leu-L-trans-4-hydroxyproline) [3].

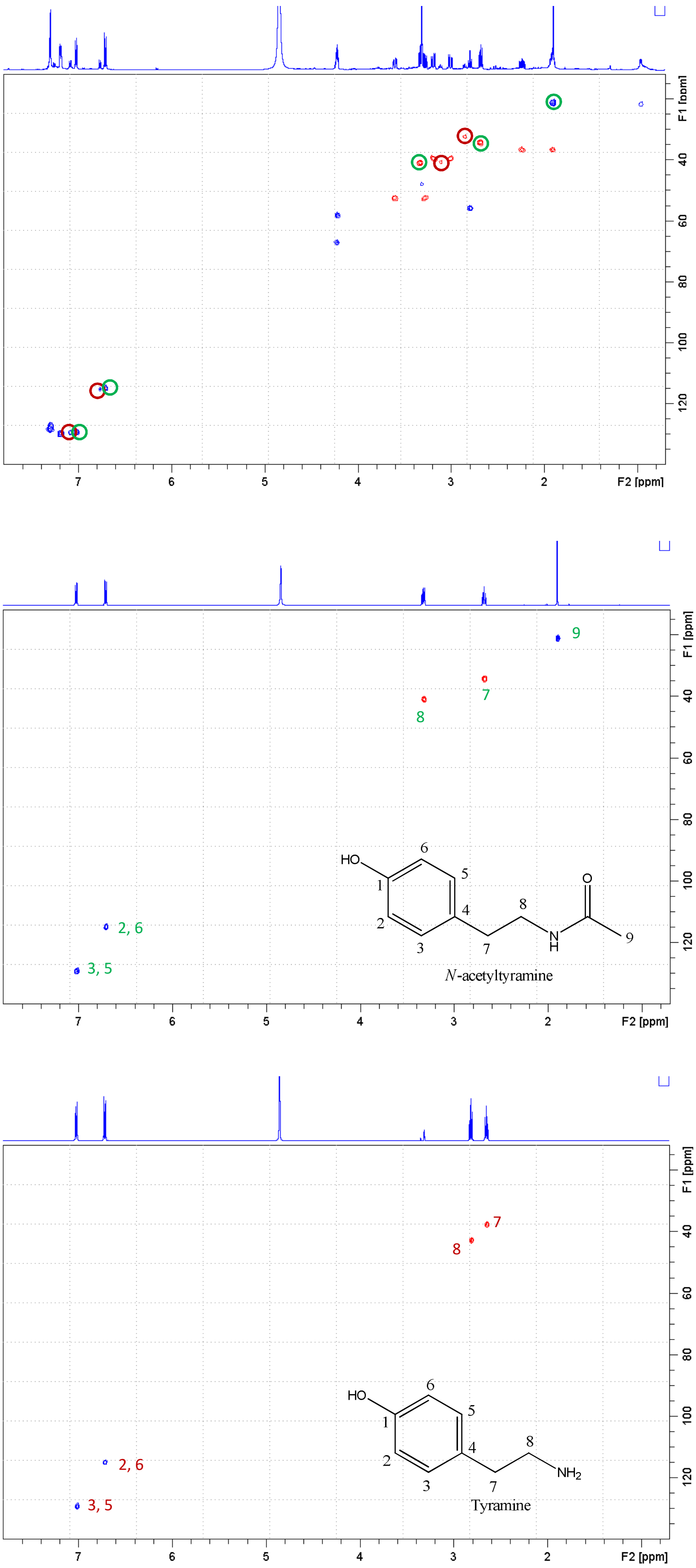

Supplementary Figure S12. HSQC spectra of QSI-active HPLC fraction 13 (upper), N-acetyltyramine standard (middle) and tyramine standard (lower). Signals from these two molecules are highlighted in the upper spectrum, where the non-highlighted signals correspond to cyclo-(D-Phe-L-trans-4-hydroxyproline)[4]

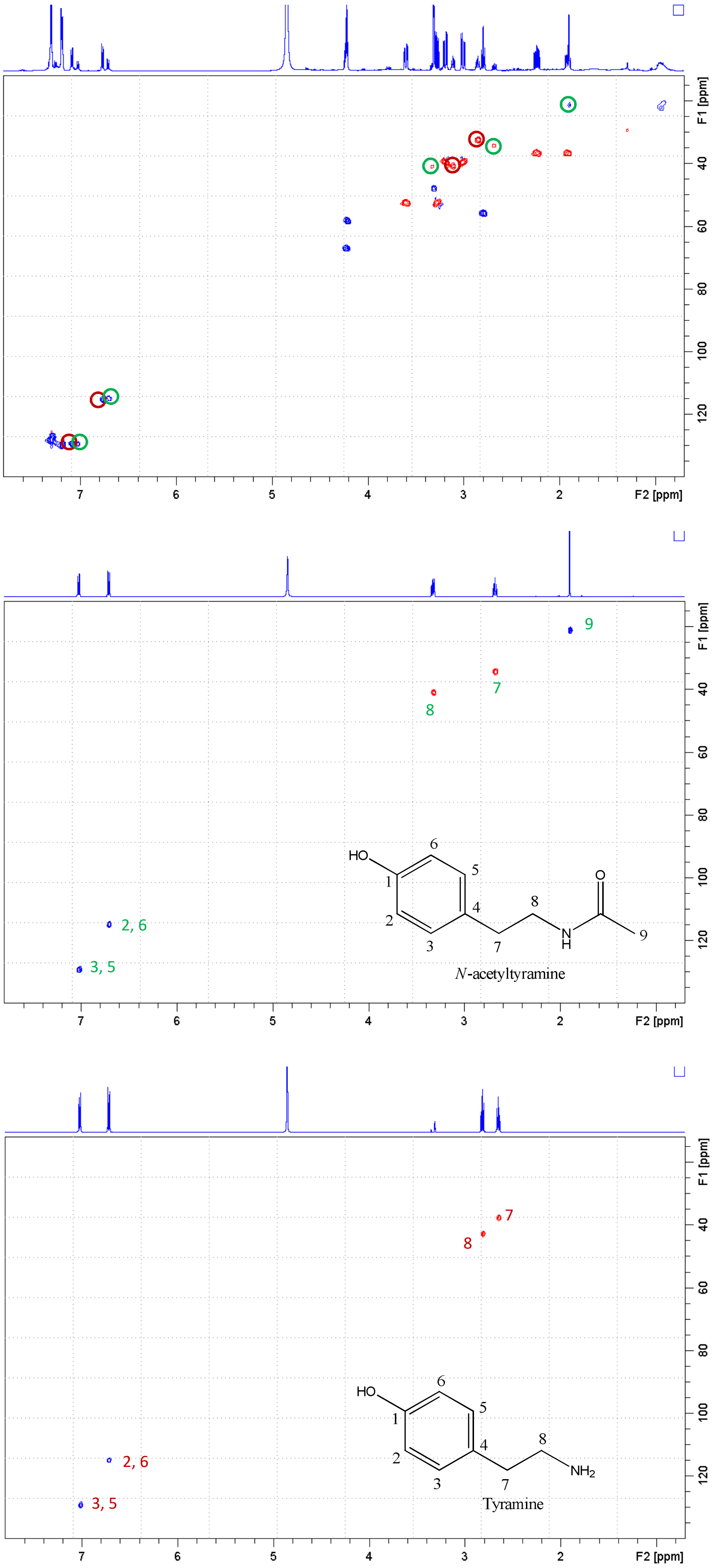

Supplementary Figure S13. HSQC spectra of QSI-active HPLC fraction 13 (upper), N-acetyltyramine standard (middle) and tyramine standard (lower). Signals from these two molecules are highlighted in the upper spectrum, where the non-highlighted signals correspond to cyclo-(D-Phe-L-trans-4-hydroxyproline) [4].

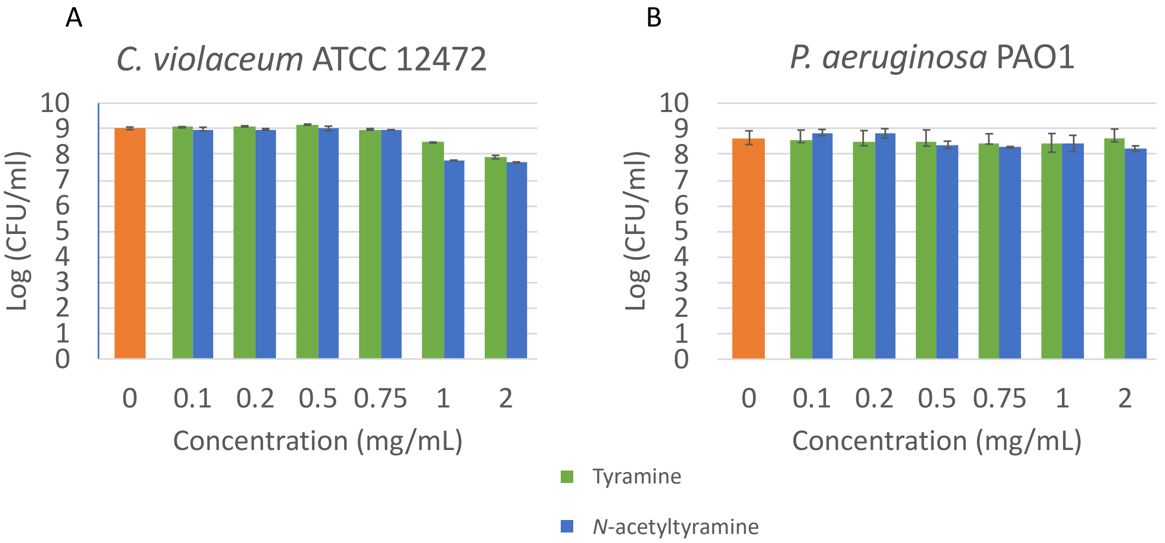

Figure S14. Growth of *C. violaceum* ATCC 12472 (A) and *P. aeruginosa* PAO1 (B) in the absence and presence of different concentrations of tyramine and *N*-acetyltyramine. Bacterial growth was measured by colony counting onto LB plates after incubating the bacteria in each condition for 24h. Data are presented as the logarithm of mean CFU ± SD, n=3.

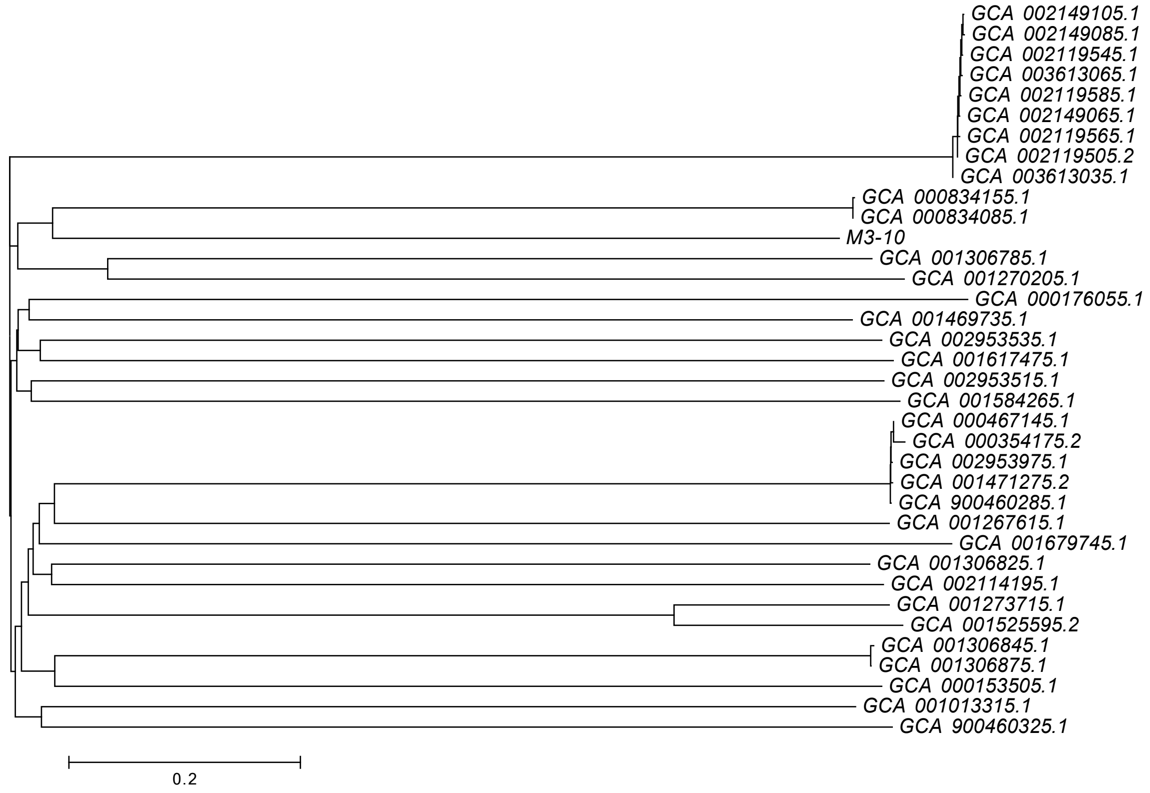

Figure S15. Neighbor-Joining distance clustering tree based on the ANI values between M3-10 and the rest of the *Vibrio alginolyticus* genomes available in NCBI.

Supplementary Table S1. NMR assignment (protonated carbons) of tyramine and *N*-acetyltyramine signals observed in the three QSI-active HPLC fractions (500 MHz, CD3OD).

	Tyramine		*N*-acetyltyramine	
Position	δ_C	δ_H (*J* in Hz)	δ_C	δ_H (*J* in Hz)
2, 6	115.3	6.77, d (8.4)	114.9	6.71, d (8.4)
3, 5	129.3	7.08, d (8.4)	129.3	7.02, d (8.4)
7	32.4	2.85, t (7.3)	34.3	2.68, t (7.4)
8	40.7	3.11, t (7.4)	41.0	3.33, t (7.4)
9	-	-	21.1	1.90, s

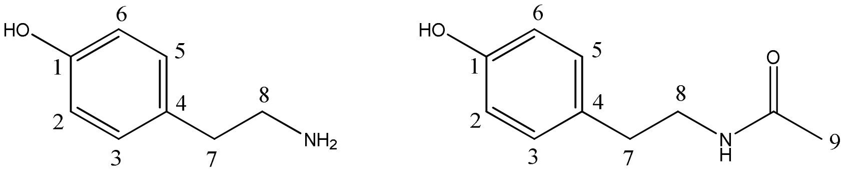

Tyramine   *N*-acetyltyramine

Supplementary Table S2. NMR assignment (protonated carbons) of tyramine and *N*-acetyltyramine standards (500 MHz, CD3OD).

	Tyramine		*N*-acetyltyramine	
Position	δ_C	δ_H (*J* in Hz)	δ_C	δ_H (*J* in Hz)
2, 6	115.0	6.71, d (8.4)	114.9	6.71, d (8.4)
3, 5	129.3	7.01, d (8.4)	129.3	7.02, d (8.4)
7	37.7	2.65, t (7.2)	34.3	2.68, t (7.4)
8	42.9	2.81, t (7.2)	41.0	3.33, t (7.4)
9	-	-	21.1	1.90, s

Supplementary Table S3. Relative abundance (estimated by NMR) of tyramine, N-acetyltyramine and diketopiperazines in the three QSI-active HPLC fractions 12, 13 and 14. Qualitative QSI activity is also indicated (* weak activity, ** medium activity and *** high activity)

HPLC fraction	QSI activity	Tyramine	*N*-acetyltyramine	DKP1*	DKP2*
Fraction 12	**	1.0	1.6	5.0	0
Fraction 13	***	1.1	3.5	0	4.1
Fraction 14	*	1.1	0.4	0	3.9

*DKP1= cyclo-(L-Leu-L-*trans*-4-hydroxyproline); DKP2= cyclo-(D-Phe-L-trans-4-hydroxyproline)

Supplementary Table S4. ANIb values of strain M3-10 compared to the other *V. alginolyticus* strains whose genome is available

	M3-10	GCA_000834155.1	GCA_002149105.1	GCA_001306845.1	GCA_001273715.1	GCA_002953975.1	GCA_000467145.1	GCA_001306825.1	GCA_002149085.1	GCA_002119585.1	GCA_001306875.1	GCA_000153505.1	GCA_000176055.1	GCA_000834085.1	GCA_002149065.1	GCA_900460285.1	GCA_900460325.1	GCA_002119565.1	GCA_002953535.1	GCA_001471275.2	GCA_001013315.1	GCA_001306785.1	GCA_002119545.1	GCA_002953515.1	GCA_001469735.1	GCA_001584265.1	GCA_001270205.1	GCA_003613035.1	GCA_002114195.1	GCA_002119505.2	GCA_001679745.1	GCA_001525595.2	GCA_000354175.2	GCA_001267615.1	GCA_003613065.1	GCA_001617475.1
M3-10	100.000	98.631	98.456	98.541	98.554	98.551	98.541	98.549	98.469	98.455	98.556	98.540	98.441	98.624	98.475	98.566	98.499	98.476	98.503	98.514	98.571	98.542	98.469	98.525	98.564	98.471	98.531	98.499	98.519	98.477	98.466	98.508	98.555	98.576	98.469	98.495
GCA_000834155.1		100.000	98.408	98.501	98.511	98.517	98.540	98.490	98.420	98.418	98.515	98.517	98.446	99.999	98.438	98.507	98.508	98.410	98.525	98.521	98.543	98.523	98.435	98.533	98.507	98.510	98.530	98.447	98.525	98.411	98.459	98.549	98.517	98.546	98.410	98.496
GCA_002149105.1			100.000	98.429	98.416	98.419	98.421	98.406	99.998	99.996	98.415	98.417	98.356	98.429	99.996	98.419	98.474	99.997	98.404	98.425	98.408	98.421	99.998	98.413	98.460	98.398	98.416	99.997	98.439	99.997	98.380	98.409	98.388	98.407	99.997	98.418
GCA_001306845.1				100.000	98.500	98.549	98.534	98.521	98.422	98.419	99.997	98.573	98.432	98.513	98.431	98.519	98.486	98.424	98.523	98.513	98.550	98.537	98.425	98.507	98.515	98.483	98.465	98.452	98.490	98.426	98.494	98.513	98.525	98.488	98.431	98.477
GCA_001273715.1					100.000	98.521	98.523	98.557	98.412	98.407	98.508	98.482	98.424	98.511	98.407	98.519	98.502	98.420	98.479	98.523	98.515	98.479	98.420	98.483	98.530	98.482	98.431	98.420	98.532	98.426	98.442	99.616	98.533	98.497	98.396	98.453
GCA_002953975.1						100.000	99.997	98.508	98.420	98.427	98.490	98.530	98.400	98.536	98.393	99.997	98.465	98.429	98.507	99.998	98.512	98.492	98.414	98.506	98.521	98.478	98.443	98.389	98.525	98.407	98.459	98.510	99.990	98.567	98.431	98.471
GCA_000467145.1							100.000	98.517	98.418	98.411	98.558	98.523	98.405	98.519	98.427	99.997	98.476	98.406	98.486	99.997	98.514	98.487	98.407	98.497	98.521	98.472	98.478	98.438	98.549	98.406	98.457	98.514	99.994	98.555	98.407	98.477
GCA_001306825.1								100.000	98.414	98.416	98.552	98.508	98.436	98.495	98.431	98.499	98.484	98.437	98.526	98.475	98.511	98.542	98.422	98.524	98.502	98.505	98.495	98.440	98.574	98.432	98.471	98.550	98.499	98.532	98.409	98.492
GCA_002149085.1									100.000	99.997	98.418	98.426	98.355	98.415	99.998	98.422	98.459	99.997	98.419	98.434	98.420	98.422	99.997	98.404	98.452	98.411	98.400	99.998	98.444	99.999	98.389	98.391	98.380	98.378	99.998	98.397
GCA_002119585.1										100.000	98.434	98.436	98.351	98.404	99.997	98.437	98.473	99.997	98.416	98.412	98.419	98.428	99.998	98.405	98.467	98.414	98.412	99.996	98.448	99.998	98.392	98.404	98.401	98.382	99.997	98.424
GCA_001306875.1											100.000	98.584	98.398	98.520	98.433	98.538	98.487	98.437	98.515	98.528	98.547	98.551	98.416	98.518	98.519	98.494	98.462	98.436	98.509	98.428	98.490	98.510	98.530	98.512	98.416	98.467
GCA_000153505.1												100.000	98.416	98.529	98.426	98.501	98.502	98.439	98.487	98.492	98.516	98.524	98.431	98.487	98.547	98.477	98.447	98.419	98.555	98.436	98.443	98.499	98.513	98.507	98.406	98.476
GCA_000176055.1													100.000	98.459	98.375	98.396	98.409	98.360	98.400	98.416	98.420	98.427	98.361	98.411	98.477	98.428	98.397	98.380	98.413	98.380	98.341	98.420	98.410	98.416	98.355	98.450
GCA_000834085.1														100.000	98.427	98.531	98.524	98.403	98.537	98.529	98.555	98.516	98.444	98.531	98.513	98.524	98.535	98.442	98.514	98.413	98.450	98.537	98.512	98.512	98.422	98.500
GCA_002149065.1															100.000	98.387	98.473	99.995	98.430	98.410	98.404	98.436	99.997	98.402	98.450	98.392	98.405	99.998	98.432	99.997	98.389	98.397	98.402	98.420	99.996	98.422
GCA_900460285.1																100.000	98.474	98.434	98.492	99.997	98.515	98.489	98.428	98.501	98.515	98.463	98.454	98.390	98.547	98.446	98.464	98.506	99.990	98.557	98.428	98.470
GCA_900460325.1																	100.000	98.452	98.527	98.461	98.561	98.439	98.471	98.470	98.507	98.476	98.419	98.469	98.490	98.459	98.432	98.468	98.459	98.455	98.466	98.473
GCA_002119565.1																		100.000	98.426	98.430	98.428	98.420	99.997	98.405	98.439	98.419	98.414	99.995	98.453	99.998	98.398	98.385	98.392	98.384	99.996	98.409
GCA_002953535.1																			100.000	98.508	98.517	98.487	98.399	98.503	98.523	98.485	98.459	98.448	98.497	98.420	98.464	98.478	98.475	98.488	98.405	98.535
GCA_001471275.2																				100.000	98.538	98.504	98.427	98.487	98.513	98.481	98.457	98.410	98.533	98.426	98.466	98.514	99.988	98.548	98.421	98.466
GCA_001013315.1																					100.000	98.529	98.410	98.505	98.536	98.516	98.523	98.408	98.504	98.426	98.452	98.542	98.505	98.527	98.404	98.506
GCA_001306785.1																						100.000	98.410	98.506	98.507	98.514	98.651	98.456	98.527	98.404	98.431	98.475	98.459	98.466	98.414	98.504
GCA_002119545.1																							100.000	98.405	98.459	98.406	98.392	99.996	98.458	99.998	98.369	98.402	98.379	98.386	99.996	98.426
GCA_002953515.1																								100.000	98.532	98.512	98.481	98.431	98.490	98.398	98.410	98.478	98.479	98.496	98.408	98.490
GCA_001469735.1																									100.000	98.512	98.530	98.439	98.505	98.454	98.467	98.504	98.525	98.507	98.481	98.536
GCA_001584265.1																										100.000	98.469	98.401	98.434	98.439	98.368	98.480	98.473	98.473	98.434	98.485
GCA_001270205.1																											100.000	98.422	98.482	98.412	98.422	98.447	98.470	98.457	98.411	98.466
GCA_003613035.1																												100.000	98.446	99.998	98.381	98.398	98.408	98.426	99.997	98.430
GCA_002114195.1																													100.000	98.466	98.496	98.446	98.521	98.524	98.459	98.464
GCA_002119505.2																														100.000	98.396	98.389	98.403	98.391	99.998	98.405
GCA_001679745.1																															100.000	98.455	98.438	98.488	98.382	98.431
GCA_001525595.2																																100.000	98.495	98.467	98.384	98.443
GCA_000354175.2																																	100.000	98.529	98.397	98.485
GCA_001267615.1																																		100.000	98.422	98.520
GCA_003613065.1																																			100.000	98.398
GCA_001617475.1																																				100.000

Color key
> 98
> 98.5
> 99

Supplementary Table S5. Protein sequence accession numbers for tyrosine decarboxylases used in the BLASTp homologue search.

1. CAI39170.2_1	2. CAI39169.2_1	3. AFP73381.1_1
4. AGW24520.1_1	5. AGW24519.1_1	6. BAE02560.1_1
7. ACS15340.1_1	8. ACS15339.1_1	9. ACS15338.1_1
10. CAY83536.1_1	11. CAY83534.1_1	12. CAY72357.1_1
13. CAY72352.1_1	14. CAY72349.1_1	15. CAY72347.1_1
16. CAY72344.1_1	17. CAY72343.1_1	18. CAY72341.1_1
19. CAY72338.1_1	20. CAY72336.1_1	21. CAY72335.1_1
22. CAY72333.1_1	23. CAY72330.1_1	24. BAE02559.1_1
25. AWT58522.1_1	26. AWT58521.1_1	27. AWT58520.1_1
28. AWT58519.1_1	29. AWT58518.1_1	30. AWT58517.1_1
31. AWT58516.1_1	32. BAI67125.1_1	33. WP_135180232.1_1
34. WP_135017352.1_1	35. E4V23_RS18420	36. WP_135043646.1_1
37. E4V46_RS09510	38. TFU21072.1_3	39. TFU11427.1_1
40. TFT82455.1_1	41. TFT56894.1_1	42. TFT37476.1_1
43. EVY62_RS19255	44. WP_129949649.1_1	45. WP_088207211.1_1
46. RZA37683.1_1	47. RZA37564.1_3	48. RYH14358.1_1
49. BHE89_RS17365	50. H049_RS0124195	51. H049_RS0123705
52. EBQ16_RS07935	53. WP_121909587.1_1	54. EA135_RS16540
55. RLZ56634.1_1	56. RLZ55416.1_1	57. C3O82_RS13145
58. RBH48265.1_1	59. RBH41953.1_1	60. WP_017628132.1_1
61. PSQ86773.1_1	62. PSQ85593.1_4	63. BRD33_05025
64. CUS67_RS12755	65. WP_104877381.1_2	66. CUM70_RS13060
67. CUM70_RS13050	68. PQF68676.1_2	69. CUS67_12755
70. PQD88208.1_1	71. PQD88205.1_1	72. C4A96_RS26915
73. C4A96_RS06470	74. C4A96_RS04445	75. C4A96_RS04265
76. PKN32413.1_1	77. CQR37_RS16425	78. PHL20111.1_1
79. OUK41848.1_2	80. OUK40079.1_2	81. OSP71327.1_1
82. OSP67774.1_2	83. WP_071425714.1_1	84. WP_080389721.1_2
85. WP_011109458.1_1	86. OIK52589.1_1	87. BHE89_18525
88. KXF72672.1_2	89. KRM71154.1_1	90. EAO52030.1_1
91. CBW46640.1_1	92. ABC68277.1_1	93. CAH04395.1_1
94. SCD69325.1_2		

Supplementary methods S1. *Identification of QSI compounds in active HPLC fractions using LC-DAD-HRMS and NMR analyses.*

After LC-DAD-HRMS analyses, dereplication against our in-house databases [1] identified the presence of both tyramine and *N*-acetyltyramine (Figure 1) in each of the three bioactive HPLC fractions (Figure S3). On the other hand, NMR signals from two different compounds compatible with those reported for tyramine [5] and *N*-acetyltyramine [5,6] were present in all three fractions, while signals corresponding to other compounds (diketopiperazines) were only observed in one or two of the three bioactive fractions (see Figures S4-S6). Interpretation of 2D NMR spectra (COSY, HSQC and HMBC; data not shown) confirmed the dereplication of tyramine and *N*-acetyltyramine in the three fractions and established the presence of cyclo-(L-Leu-L-trans-4-hydroxyproline) in fraction 12 [3] and cyclo-(D-Phe-L-trans-4-hydroxyproline) in fractions 13 and 14 [4]. Additionally, the identity of tyramine and *N*-acetyltyramine was definitively confirmed by directly comparing the NMR spectra (1H and HSQC) of the three bioactive fractions with the spectra of the standards of these two related compounds acquired in the same spectrometer (see Supplementary Figures S7-S13 and Supplementary Tables S4 and S5). The relative abundance of tyramine, *N*-acetyltyramine and diketopiperazines in the three fractions was easily established from the 1H NMR spectra (see Table S1), clearly indicating that either tyramine, *N*-acetyltyramine or both are the QSI compounds produced by the *V. alginolyticus* M3-10 strain. A QSI activity test carried out with pure standards of these two related molecules later confirmed that both are indeed QSI compounds.

Supplementary material references

1. Pérez-Victoria, I.; Martín, J.; Reyes, F. Combined LC/UV/MS and NMR Strategies for the Dereplication of Marine Natural Products. *Planta Med.* **2016**, *82*, 857–871.
2. Martín, J.; Crespo, G.; González-Menéndez, V.; Pérez-Moreno, G.; Sánchez-Carrasco, P.; Pérez-Victoria, I.; Ruiz-Pérez, L.M.; González-Pacanowska, D.; Vicente, F.; Genilloud, O.; et al. MDN-0104, an Antiplasmodial Betaine Lipid from *Heterospora chenopodii*. *J. Nat. Prod.* **2014**, *77*, 2118–2123.
3. Rustamova, N.; Bobakulov, K.; Begmatov, N.; Turak, A.; Yili, A.; Aisa, H.A. Secondary metabolites produced by endophytic *Pantoea ananatis* derived from roots of *Baccharoides anthelmintica* and their effect on melanin synthesis in murine B16 cells. *Nat. Prod. Res.* **2019**, 1–6.
4. Ye, X.; Chai, W.; Lian, X.-Y.; Zhang, Z. Novel propanamide analogue and antiproliferative diketopiperazines from mangrove *Streptomyces* sp. Q24. *Nat. Prod. Res.* **2017**, *31*, 1390–1396.
5. Sun, J.F.; Wu, Y.; Yang, B.; Liu, Y. Chemical Constituents of Marine Sponge *Halichondria* sp. from South China Sea. *Chem. Nat. Compd.* **2015**, *51*, 975–977.
6. Gutiérrez, M.; Capson, T.L.; Guzmán, H.M.; González, J.; Ortega-Barría, E.; Quiñoá, E.; Riguera, R. Antiplasmodial metabolites isolated from the marine octocoral Muricea austera. *J. Nat. Prod.* **2006**, *69*, 1379–1383.
